# Presence of Group A streptococcus frequently assayed virulence genes in invasive disease: a systematic review and meta-analysis

**DOI:** 10.3389/fcimb.2024.1337861

**Published:** 2024-06-03

**Authors:** Kimona Rampersadh, M. Taariq Salie, Kelin C. Engel, Clinton Moodley, Liesl J. Zühlke, Mark E. Engel

**Affiliations:** ^1^ AFROStrep Research Group, Department of Medicine and Cape Heart Institute, University of Cape Town, Cape Town, South Africa; ^2^ Department of Pathology, Division of Medical Microbiology, Faculty of Health Sciences, University of Cape Town, Cape Town, South Africa; ^3^ The National Health Laboratory Service, Microbiology, Groote Schuur Hospital, Cape Town, South Africa; ^4^ Division of Paediatric Cardiology, Department of Paediatrics, Faculty of Health Sciences, University of Cape Town, Cape Town, South Africa; ^5^ South African Medical Research Council, Parrow Valley, Cape Town, South Africa

**Keywords:** Group A streptococcus, *Streptococcus pyogenes*, invasive disease, virulence factors, superantigens

## Abstract

**Introduction:**

It is currently unclear what the role of Group A streptococcus (GAS) virulence factors (VFs) is in contributing to the invasive potential of GAS. This work investigated the evidence for the association of GAS VFs with invasive disease.

**Methods:**

We employed a broad search strategy for studies reporting the presence of GAS VFs in invasive and non-invasive GAS disease. Data were independently extracted by two reviewers, quality assessed, and meta-analyzed using Stata®.

**Results:**

A total of 32 studies reported on 45 putative virulence factors [invasive (n = 3,236); non-invasive (n = 5,218)], characterized by polymerase chain reaction (PCR) (n = 30) and whole-genome sequencing (WGS) (n = 2). The risk of bias was rated as low and moderate, in 23 and 9 studies, respectively. Meta-,analyses of high-quality studies (n = 23) revealed a significant association of *speM* [OR, 1.64 (95%CI, 1.06; 2.52)] with invasive infection. Meta-analysis of WGS studies demonstrated a significant association of *hasA* [OR, 1.91 (95%CI, 1.36; 2.67)] and *speG* [OR, 2.83 (95%CI, 1.63; 4.92)] with invasive GAS (iGAS). Meta-analysis of PCR studies indicated a significant association of *speA* [OR, 1.59 (95%CI, 1.10; 2.30)] and *speK* [OR, 2.95 (95%CI, 1.81; 4.80)] with invasive infection. A significant inverse association was observed between *prtf1* [OR, 0.42 (95%CI, 0.20; 0.87)] and invasive infection.

**Conclusion:**

This systematic review and genomic meta-analysis provides evidence of a statistically significant association with invasive infection for the *hasA* gene, while *smeZ*, *ssa*, *pnga3*, *sda1*, *sic*, and *NaDase* show statistically significantly inverse associations with invasive infection. *SpeA*, *speK*, and *speG* are associated with GAS virulence; however, it is unclear if they are markers of invasive infection. This work could possibly aid in developing preventative strategies.

## Introduction

Group A streptococcus (GAS) is responsible for a range of disease, causing both superficial and invasive disease (Tapiainen et al., 2016; Espadas-Maciá et al., 2018; CDC, 2022). GAS invasive disease is characterized by the isolation of strains from normally sterile sites in the body, e.g., blood, cerebrospinal fluid, pleural fluid, joint fluid, pericardial fluid, or peritoneal fluid, or non-sterile sites such as wounds associated with necrotizing fasciitis (NF) and streptococcal toxic shock syndrome (STSS). Where GAS strains are isolated from patients with pharyngitis, impetigo, scarlet fever, and erysipelas, the disease is regarded as non-invasive/superficial. Since 2005, the global burden from invasive GAS diseases is reported to be approximately 517,000 deaths with figures disproportionately higher in developing countries as compared to those in developed countries (Carapetis et al., 2005).

GAS are genetically diverse, with various complements of virulence factors that engage a vast variety of host defenses (Walker et al., 2014). Among virulence factors associated with the pathogenesis of GAS, the M protein and streptococcal pyrogenic exotoxins (Spes) are the major ones (Shannon et al., 2019). In addition, GAS produces surface proteins, known as adhesins, including pilli (Spy0130, Spy0128, Cpa), fibronectin-binding proteins (PrtF1, PrtF2, SfbI, SfbII, SOF, Fbaa, and Fbab), collagen-like proteins (Scl1, Scl2), laminin-binding proteins (Lbp, Shr), and plasminogen-binding proteins (GAPDH, SEN), which have also been reported (Walker et al., 2014). GAS also produces numerous secreted factors, such as streptokinase (Ska), engaged in interactions with complements; streptolysin S (SLS), a promoter involved in fibrinolysis and neutrophil modulation; and hyaluronidase and cysteine proteinases, which are often considered to be virulence factors (Walker et al., 2014; Barnett et al., 2015).

The M protein is a key surface virulence factor encoded by the *emm* gene, which displays marked variability in the 5' hypervariable region and forms the basis for *emm* genotyping (DebRoy et al., 2018). To date, in excess of 250 different *emm types* have been reported (Sanderson-Smith et al., 2014). M protein is associated with several stages in GAS pathogenesis, namely, adhesion, internalization, evasion of the immune system, and tissue invasion. The contribution of the M protein to virulence is attributed to immune modulatory effects, mediated by the binding of host proteins such as immunoglobulins and fibrinogen, as well as providing antiphagocytic functions critical for GAS survival in tissues and bodily fluids (Smeesters et al., 2010). In an effort to predict the basic genetic features of GAS isolates, Sanderson-Smith et al. introduced a cluster-based classification for GAS (Sanderson-Smith et al., 2014). This system classifies *emm types* into clusters that have the same or similar sequences as well as host binding properties, allowing for previously characterized GAS *emm types* to be classified into 48 *emm clusters*, complementing the *emm typing* scheme, which may assist in improving studies associated with M protein function, epidemiological surveillance, GAS virulence determinants, and therapeutic developments such as vaccines (Sanderson-Smith et al., 2014).

Spes are secreted proteins displaying the traits of superantigens (SAgs), which putatively play a role in the pathogenesis of invasive infections. Superantigens or exotoxins have thus far been described as the most potent proteins involved in stimulating T-cell proliferation and differentiation. Superantigens have the ability to circumvent the usual antigen processing and presentation by cross-linking MHC class II molecules and the V*β* region of the antigen receptor on a subset of T lymphocytes (Fraser and Proft, 2008; Zeppa et al., 2017), leading to T-cell proliferation. This induces a huge secretion of inflammatory cytokines (Herman et al., 1991). Overproduction of these cytokines can lead to shock, tissue damage, and organ failure. There have been more than 40 bacterial superantigens reported in the literature, of which 12 distinct extracellular superantigens have been elucidated in GAS, which include Spes (A, C, G, H, I, J, K, L, M), streptococcal mitogenic exotoxins (*smeZ*) 1 and 2, and the streptococcal superantigen (*ssa*) (Proft and Fraser, 2003; Commons et al., 2008; Berman et al., 2014; Reglinski et al., 2019). Superantigens implicated in GAS virulence have been associated with diseases such as scarlet fever, STSS, and rheumatic fever (Barnett et al., 2015). *Emm types* have been reported to be associated with specific superantigens, and these associations vary in GAS populations collected from various geographical locations (Commons et al., 2008).

GAS cell surface proteins include various adhesins, which allows for bacterial–host interactions, permitting GAS colonization to diverse tissues in the human body (Walker et al., 2014). GAS surface proteins use three known mechanisms to attach to the bacterial surface, namely, covalent binding to the peptidoglycan through a C-terminal LPxTG motif, which is recognized by sortase A (Barnett and Scott, 2002); covalent attachment to the cell membrane via N-terminal modifications with lipoproteins (Nobbs et al., 2009); and non-covalent binding to cell surface components (Nobbs et al., 2009). Secreted GAS virulence factors target numerous components of the immune response. The host immune response is avoided through several mechanisms, such as interference of the chemokine gradient via degradation, hindering of neutrophil migration, damaging of host cells through pore-forming toxins, degradation of neutrophil extracellular traps via DNases, cleavage of circulating host effector proteins, destruction of epithelial barriers and extracellular matrix proteins, degradation of macrophage proliferation and function, and evading of intracellular activities once inside the host (Barnett et al., 2022).

Given that there are currently no syntheses of existing studies, we sought to provide an evidence-based assessment, from published articles, of the key virulence factors associated with invasive GAS infection. We envisaged that the results of this study will serve to inform further research addressing the role of GAS virulence factors in both invasive and non-invasive GAS infections.

## Materials and methods

This systematic review was prepared according to the Preferred Reporting Items for Systematic Reviews and Meta-Analyses protocols (Moher et al., 2009).

### Review question

This systematic review sought to identify the genomic elements associated with invasive GAS infection. Using the PEO (population, exposure, and outcome) mnemonic, where P refers to children or adults, E to GAS virulence factors, and outcome to invasive disease, the review question was, *Are specific GAS virulence genes associated with invasive disease in patients with GAS-associated infection?*

### Search strategy

To maximize sensitivity, a broad search strategy was designed. The main search included individual searches using Medical Subject Headings (MeSH). A combination of terms relating to “invasive”, “virulence”, and “pathogenic” were used (**Supplementary Table S1**—available at https://doi.org/10.25375/uct.23708346). The search was carried out, independently, by two reviewers among several databases including Medline (accessed via PubMed), Scopus, and Web of Science from the earliest published data to 19 July 2023. Search results were complemented with snowballing searches in Google Scholar, thesis databases, and conference proceedings and scanning the reference lists of the articles. The search strategy was modified to suit the vocabulary of individual database(s). The search was not restricted by language or date of publication.

### Inclusion criteria

We included studies reporting sequencing of the genetic elements associated with invasive and non-invasive GAS infection across all age groups, ethnicities, and socioeconomic and educational backgrounds, globally. Invasive infection was broadly defined as recovery of GAS isolates from normally sterile sites with samples, including cerebrospinal fluid (CSF), blood, and synovial and pleural fluids. We considered published articles; all study designs were considered for inclusion. In addition, articles published in other languages with complete English abstracts were considered. Studies incorporating polymerase chain reaction (PCR)/whole-genome sequencing (WGS) were prioritized, given the superiority of these methods in producing molecular sequence data (Chochua et al., 2017; Plainvert et al., 2018).

### Exclusion criteria

We excluded opinion pieces, letters, narrative reviews, and any other publications lacking primary data and/or unambiguous method descriptions. Where publications utilized the same data, the most recent and complete versions were considered.

### Data extraction and management

Search results from all aforementioned databases and reference search results were managed with the EndNote referencing software. A data extraction form was compiled, which included predefined criteria. Data extraction was conducted by KR and verified by a second reviewer (KE) and a third reviewer (TS).

### Quality assessment

The internal and external validity and generalizability of the included study results were evaluated for risk of bias. An assessment of the risk of bias informed the evaluation of heterogeneity in the pooled analysis. A quality assessment tool for evaluating prevalence studies as suggested by Hoy and colleagues (and adapted by Salie et al.) was adapted for the purpose of this review; the revised version allows for a composite score to assist with a relative comparison between the studies, thereby reducing reviewers’ subjectivity (Salie et al., 2020). Briefly, Salie et al. added a quantitative scoring system to the risk of bias table, allocating four points for external validly score and six points for internal validity. Six domains were considered for this review. The scoring system tool classifies studies into different categories based on their overall scores: high risk if the score is 1–2 points, moderate risk for 3–4 points, and low risk if it falls within the range of 5–6 points.

### Statistical analysis

We conducted statistical analyses using Stata version 14.1 (Stata Corp., College Station, TX, USA) to determine the overall the effect size (odds ratio and 95%CI) of association between virulence factors and invasive GAS disease. Meta-analyses are presented by tables. Where a meta-analysis was not feasible, because data were either too heterogeneous or insufficient to allow for meaningful pooling, we compiled a narrative report of the results.

## Results

### Study selection

**Figure 1:**
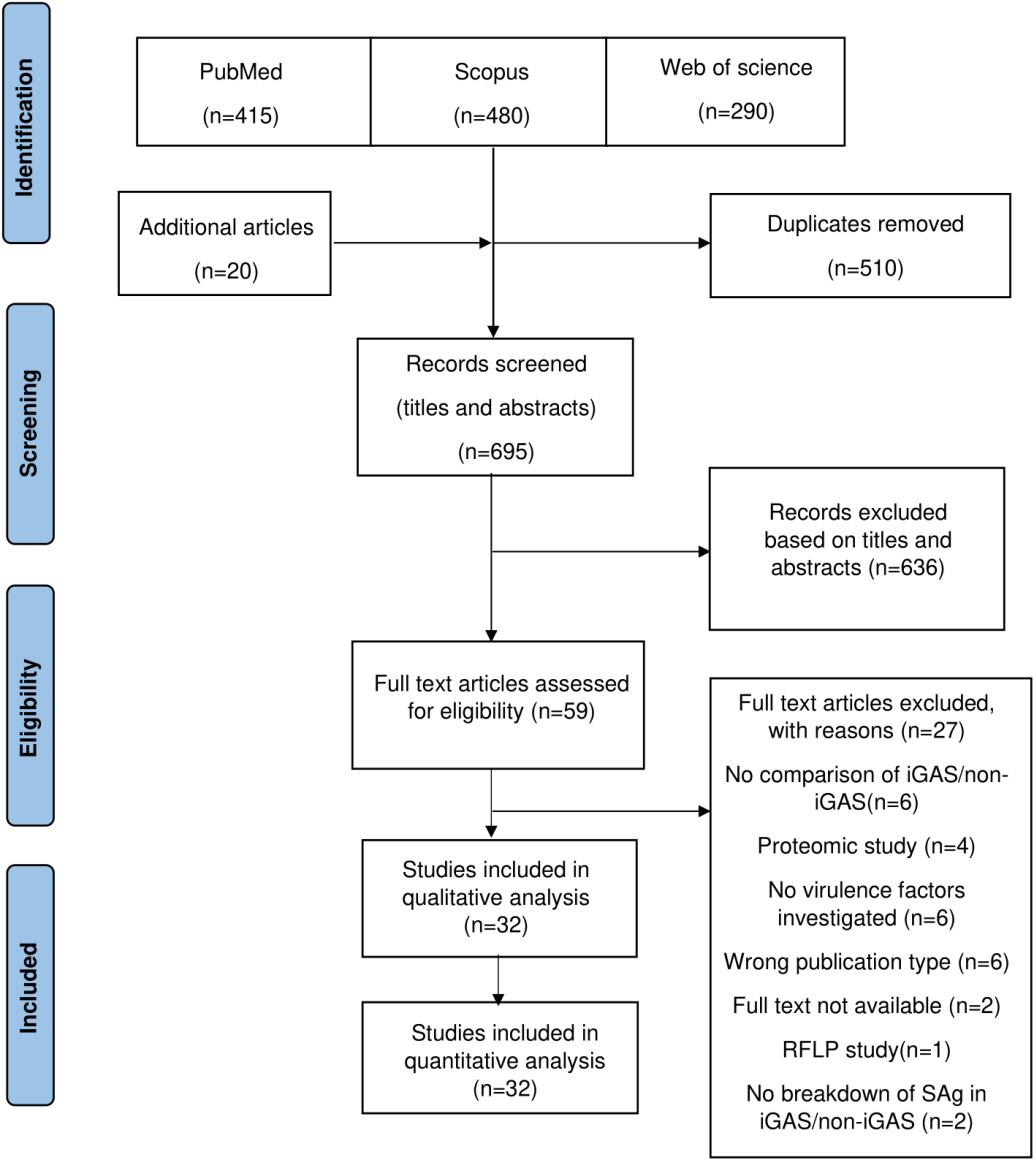
Preferred Reporting Items for systematic reviews and meta-analyses (PRISMA) flow diagram.

The literature search identified 1,185 articles for consideration for inclusion from the respective electronic databases (**Figure 1**). Following deduplication and handsearching, 695 articles were subjected to screening of titles and abstracts, of which 59 articles required full-text review. Finally, 32 articles met the inclusion criteria and were included in the review. A single restriction fragment length polymorphism (RFLP) study was excluded since this review only included sequence-based methods. A detailed list of the excluded studies is documented in **Supplementary Table S2** (available at https://doi.org/10.25375/uct.23708346).

### Study characteristics

The study characteristics of included studies are presented in **Table 1**. The included articles comprised molecular studies, published between 1992 and 2022, reporting on the association of GAS infection with descriptions of genetic elements (invasive, n = 3,236; non-invasive, n = 5,218). The 32 articles reported in this systematic review comprised 33 datasets from 22 countries, namely, Poland (1 article), USA (2), Italy (3), Belgium (2), China (2), Germany (2), Norway (3), Sweden (2), Denmark (1), Australia (1), Bulgaria (1), Canada (1), France (1), Hong Kong (1), India (1), Ireland (1), Japan (1), Pakistan (1), Romania (1), Spain (1), Taiwan (1), and Tunisia (1); the remaining study comprised samples from 12 countries. Where invasive disease was not defined, we accepted the authors’ classification of invasiveness. Methods for detecting the virulence factors included PCR (n = 30) and WGS (n = 2).

**Table 1:** Characteristics of the included studies.

**Study ID**	**Country**	**Method**	**Sample size**	Age (years)*	**Emm type**	**Definition of invasive GAS**
Bencardino et al., 2019	Italy	PCR	Invasive (n = 5) Non-invasive (n = 116)	NS	1, 2, 4, 5, 6, 9, 11, 12, 18, 28, 29, 44, 48, 75, 78, 82, 87, 89, 118	Isolates were from normally sterile fluids (blood and pleural fluid)
Bianco et al., 2006	Italy	PCR	Invasive (n = 5) Non-invasive (n = 177)	NS	None	A few strains were isolated from subjects affected by invasive diseases (bacteremia, pneumonia, and toxic shock-like syndrome)
Chan et al., 2009*	Hong Kong	PCR	Invasive (n = 27) Non-invasive (n = 27)	53(4–100)	1, 2, 3, 4, 9, 10, 11, 12, 22, 25, 28, 42, 49, 53, 54, 58, 63, 69, 70, 73, 75, 76, 77, 81, 82, 85, 86, 87, 88, 89, 90, 93, 94, 101, 104, 106, 112, 113, 118, 124, st9505, st2904, Stg485, STMRP6	Invasive and non-invasive isolates were defined as recovery from sites that are usually sterile or non-sterile, respectively
Coppens et al., 2019	Belgium	WGS	Invasive (n = 23) Non-invasive (n = 2)	NS	1	Invasive GAS strains were randomly selected and obtained from blood and tissue [wounds/throat swabs obtained from patients with skin and soft-tissue infection (SSTI) body sites or were from an undefined origin]
Creti et al., 2005	Italy	PCR	Invasive (n = 79) Non-invasive (n = 180)	47.1 ± 23.6	1, 2, 3, 4, 5, 6, 9, 11, 12, 14, 18, 22, 27, 28, 29, 44, 50, 53, 59, 68, 75, 78, 80, 85, 87, 89, 102, 108, 110, 114, 118	NS
Darenberg et al., 2007	Sweden	Multiplex PCR	Invasive (n = 850) Non-invasive (n = 773)	68(0–99)	1, 4, 12, 28, 77, 89, 81	Invasive disease was defined by isolation of GAS from blood samples or from samples obtained from other normally sterile sites
DelVecchio et al., 2002	Australia	PCR	Invasive (n = 63) Non-invasive (n = 51)	NS	None	Patients with invasive cases of disease (necrotizing fasciitis, toxic shock syndrome, cellulitis, bacteremia)
Descheemaeker et al., 2000	Belgium	PCR	Invasive (n = 72) Non-invasive (n = 428)	NS	1, 3, 4, 6, 9, 11, 12	GAS isolates from sterile sites (blood, sterile body fluids, abscesses, or tissue) of patients with invasive infections
Ekelund et al., 2005*	Denmark	PCR	Invasive (n = 200) Non-invasive (n = 352)	(0.4–97.4)	1, 2, 3, 4, 5, 6, 9, 11, 12, 22, 28, 29, 43, 49, 58, 70, 73, 75, 76, 77, 78, 87, 88, 89, 94, 102, 105, 109, 110, 27G, st1389, st3757	Invasive GAS isolates obtained from normally sterile sites (blood, CSF, synovial fluid, pleural fluid, sterile aspirates, or tissue obtained during surgery or autopsy)
Golińska et al., 2016	Poland	PCR	Invasive (n = 30) Non-invasive (n = 37)	(18–50)	1, 2, 3, 4, 12, 28, 75, 77, 89	12 *S. pyogenes* strains originated from tissues of women with puerperal fever, and 18 strains were isolated from blood of patients with sepsis
Haukness et al., 2002	USA	PCR	Invasive (n = 17) Non-invasive (n = 63)	NS	1, 2, 3, 4, 5, 6, 12, 18, 22, 28, 59, 75, 77, 89	Invasive GAS isolates cultured from normally sterile sites of pediatric patients
Hraoui et al., 2011	Tunisia	Multiplex PCR	Invasive (n = 50) Non-invasive (n = 52)	(2–93)	1, 2, 3, 4, 6, 11, 12, 17, 18, 22, 26, 28, 33, 42, 43, 48, 59, 60, 63, 66, 67, 75, 76, 77, 81, 85, 87, 89, 92, 94, 102, 104, 106, 118, st1389, st2861UK, st3757, st432	Invasive infection was defined as the isolation of GAS from sites that are usually sterile, including blood and cerebrospinal, peritoneal, pleural, and joint fluids, and deep tissue abscesses, or from a superficial site in association with STSS or NF
Hsueh, 1998	Taiwan	PCR	Invasive (n = 44) Non-invasive (n = 28)	(2–90)	1, 6, 12	Isolates from normally sterile sites were considered invasive
Jing et al., 2006	China	PCR	Invasive (n = 10) Non-invasive (n = 76)	NS	1, 12, 8, 18, 80, 28, 101, 4, 66, 77, 94, 3, 6, 23, 44, 63, 64, 75, 85, 86, 88	Invasive isolates were exclusively from blood
Khan et al., 2020	Pakistan	PCR	Invasive (n = 41) Non-invasive (n = 33)	NS	1, 4, 28, 39, 42, 55, 58, 63, 65, 68, 75, 77, 88, 93, 104, 106	*S. pyogenes* strains were procured isolated from various clinical specimens like pus, blood, wounds, tissues, body fluids, and synovial fluid
Kittang et al., 2011	Norway	PCR	Invasive (n = 22) Non-invasive (n = 101)	NS	1, 3, 4, 12, 28, 82, 87, 89	Invasive disease was defined by isolation of GAS, GCS, or GGS from a normally sterile site, or from a non-sterile site in combination with STSS or NF
Li et al., 2022	USA	WGS	Invasive (n = 236) Non-invasive (n = 417)	(0.4–99)	1, 2, 3, 4, 6, 8, 9, 11, 12, 22, 28, 49, 59, 60, 68, 74, 75, 76, 77, 81, 82, 83, 87, 89, 92, 95, 118, 169	An iGAS disease case was defined as illness with isolation of GAS from a normally sterile site or from a wound culture accompanied by necrotizing fasciitis or STSS in a resident of the surveillance area
Lintges et al., 2010	Germany	Multiplex PCR	Invasive (n = 97) Non-invasive (n = 194)	(0–88)	1, 77, 28, 4, 12, 18, 75, 2, 3, 6, 49, 78, 22, 83, 95, 11, 81, 85, 94, 102, 44/61, 5, 9, 33, 58, 59, 7582, 89, 103, 108, 118, 29, 50, 66, 90, st3757, stns554, st1731	Patients with invasive infections (blood samples and cerebrospinal fluid sample)
Luca-Harari et al., 2008	Romania	Multiplex PCR	Invasive (n = 47) Non-invasive (n = 92)	(0–83)	1, 2, 3, 4, 5, 6, 8, 9, 12, 22, 23, 25, 28, 33, 44/61, 49, 50/62, 64, 65/69, 74, 75, 76, 77, 78, 81, 84, 87, 91, 92, 95, 100, 102, 106, 119	Invasive cases were defined by the isolation of GAS from normally sterile sites (blood, CSF, pleurae, peritoneal or joint fluid, or deep tissue), or from a superficial site, in association with NF or STSS
Maripuu et al., 2008	Sweden	PCR	Invasive (n = 54) Non-invasive (n = 37)	NS	1, 2, 3, 4, 6, 8, 12, 14, 19, 22, 28, 36, 41, 44, 49, 58, 66, 68, 73, 75, 81, 82, 84, 85, 89, 91, 93, 100	The isolates were collected from patients with invasive infections: STSS, sepsis, and erysipelas
Meehan et al., 2018	Ireland	PCR	Invasive (n = 442) Non-invasive (n = 492)	43(15–69)	1, 2, 3, 4, 5, 6, 9, 11, 12, 22, 28, 75, 76, 77, 81, 87, 89, 90	iGAS cases were based on national case definitions
Michaelsen et al., 2011	Norway	PCR	Invasive (n = 24) Non-invasive (n = 24)	NS	1, 3, 4, 6, 12, 18, 28, 77	Invasive isolates consisting of NF and STSS
Muhtarova et al., 2017	Bulgaria	Multiplex PCR	Invasive (n = 35) Non-invasive (n = 203)	NS	None	Invasive isolates: punctures from peritonsillar abscesses, middle ears and sinuses, wounds, blood culture, and cerebrospinal fluid
Murakami et al., 2002	Japan	PCR	Invasive (n = 17) Non-invasive (n = 299)	NS	1, 2, 3, 4, 6, 11, 12, 13, 18, 28, 58, 75, 87, 89	Invasive isolates were obtained from blood or an unknown location
Mylvaganam et al., 2000	Norway	PCR	Invasive (n = 22) Non-invasive (n = 20)	NS	1, 3, 6, 22, 28, 75, 78	Invasive isolates were from necrotizing fasciitis, streptococcal toxic shock syndrome, and septicemic patients without necrotizing fasciitis
Nandi et al., 2002	India	PCR	Invasive (n = 8) Non-invasive (n = 52)	(5–15)	None	NS
Plainvert et al., 2014	France	PCR	Invasive (n = 435) Non-invasive (n = 138)	(0–97)	1, 2, 3, 4, 5, 6, 8, 9, 11, 12, 18, 22, 24, 25, 27, 28, 29, 30, 32, 33, 41, 42, 43, 44, 48, 49, 50, 53, 55, 58, 59, 60, 63, 64, 65, 66, 68, 69, 71, 73, 74, 75, 76, 77, 78, 81, 82, 83, 85, 87, 88, 89, 90, 92, 93, 94, 100, 101, 102, 103, 104, 106, 108, 110, 112, 113, 116, 117, 118, 122, 124, 142, 147, 158, 168, 172, 174, 176, 179, 180, 182, 183, 187, 192, 217, 230, stG1750	GAS invasive infection was defined as the isolation of bacteria from a usually sterile site (e.g., blood, cerebrospinal fluid, bone, or joint fluid) or from samples obtained from a non-sterile site in combination with the clinical signs of NF or STSS
Rivera et al., 2006	Spain	PCR	Invasive (n = 27) Non-invasive (n = 99)	(0–91)	1, 2, 3, 4, 6, 9, 11, 12, 18, 22, 25, 28, 29, 43, 44, 49, 50, 58, 59, 63, 64, 70, 75, 77, 81, 83, 87, 89, st11014	Invasive infection was defined as the recovery of GAS from sites that are usually sterile, including blood and cerebrospinal, peritoneal, pleural, and joint fluids; deep tissue abscesses; and a superficial site in clinical association with STSS or NF
Schmitz et al., 2003 ^*^	12 European countries	PCR	Invasive (n = 153) Non-invasive (n = 25)	NS	1, 3, 12, 28	Among the 202 SENTRY isolates, 149 were blood-culture isolates, 31 were wound isolates, and 22 were pharynx isolates
Strus et al., 2017	Poland and Germany	PCR	Invasive (n = 48) Non-invasive (n = 205)	NS	1, 2, 3, 4, 6, 11, 12, 27G, 28, 32, 44, 58, 66, 73, 75, 77, 78, 81, 89, 108, 122, 159, 123	Strains were isolated from wounds and deep skin infections
Tyler et al., 1992	Canada	PCR	Invasive (n = 21) Non-invasive (n = 114)	NS	None	NS
Yu et al., 2021	China	PCR	Invasive (n = 32) Non-invasive (n = 310)	<18	1, 2, 3.1, 4, 6, 12, 22, 28, 75	The strains were isolated from blood

*Ekelund et al. reported on 200 iGAS out of 201.

^*^Schmitz et al. reported on 153 iGAS out of 239 and 25 non-iGAS out of 53.

NS, not stated. *Chan et al. used a random subset of the original 285 GAS isolates. **Age reported as per the publication. Brackets denote min–max range.

*Luca-Harari et al. reported on 47 iGAS and 92 non-iGAS, as seen in **Table 2** of the article.

*Meehan et al. reported on 442 iGAS out of 473 and 492 non-iGAS out of 517.

### Assessment of risk of bias of the included studies

The risk of bias (ROB) was assessed using the Hoy criteria as modified by Salie et al. (2020). The risk of bias was rated as low and moderate in 23 and 9 studies, respectively. Clinical phenotypes were clearly defined in the majority of studies. Considering the six domains relating to our review, most studies were assessed as having a moderate to low risk of bias (**Supplementary Table S3**—available at https://doi.org/10.25375/uct.23708346), and one study lacked clarity for assessing the risk of bias (Muhtarova et al., 2017). The sampling frame for all, but one study (Golińska et al., 2016), was a true or close representation of the target population. The data collected from all included studies were directly from participants rather than through a proxy, verifying the reliability of the sample collected. The participants of the included studies were clearly described, providing adequate control definition. Both the study instrument used to measure the parameter of interest and the mode of data collection used were well described.

### Emm types

*Emm types* (n = 119) were provided in 27 studies. For interest, we list the emm types documented according to type of infection. A total of 25 studies provided data on the *emm types* in invasive and non-invasive isolates. The most prevalent *emm types* (n = 22) are presented in **Supplementary Figure S1**—available at https://doi.org/10.25375/uct.23708346; *emm1* and *emm12* were found in 25 and 22 studies, respectively. A total of 12 *emm types* (*emm1*, *emm3*, *emm11*, *emm27*, *emm49*, *emm76*, *emm81*, *emm82*, *emm83*, *emm89*, *emm90*, and *emm92*) were significantly associated with invasive GAS infection, while 6 *emm types* (*emm2*, *emm4*, *emm6*, *emm12*, *emm77*, and *emm104*) were inversely associated with invasive GAS infection (**Supplementary Table S4**—available at https://doi.org/10.25375/uct.23708346).

### Virulence factors

A total of 32 studies (n = 8,454 isolates) were amenable to meta-analysis. We found 45 different virulence elements among the studies (**Supplementary Table S5**—available at https://doi.org/10.25375/uct.23708346). When pooling the data from high-quality studies (any method), the only two genes with a significant associations with invasive disease were *speM* [8 studies, n = 1,758; OR, 1.64 (95%CI, 1.06; 2.52), I^2 =^ 24.7] and *prtf1* [3 studies, n = 424; OR, 0.42 (95%CI, 0.20; 0.87), I^2 =^ 0%], of which only *ptrf1* had a significant association when analyzed with data coming from only one method (PCR) (**Table 2**). The other two genes with significant association with invasive GAS (iGAS) using data obtained with the PCR method, *speA* [20 studies, n = 3,571; OR, 1.59 (95%CI, 1.10; 2.30), I^2 =^ 64.4%] and *speK* [4 studies, n = 840; OR, 2.95 (95%CI, 1.81; 4.80), I^2 =^ 0%], were found to be inversely associated with iGAS in the WGS study. Moreover, most of the genes identified by the WGS method came from a single study (**Supplementary Table S6**—available at https://doi.org/10.25375/uct.23708346).

**Table 2:** Meta-analyses of the association of virulence factors and invasive infection (low ROB).

Virulence factor	No. of studies	Method	No. of iGAS	No. of non-iGAS	Pooled OR	95%CI	Heterogeneity (I^2^) %	Studies used (reference no.)
*speA*	21		442/1,184	878/3,038	1.48	0.99;2.20	73.8	(Tyler et al., 1992; Hsueh et al., 1998; Descheemaeker et al., 2000; Mylvaganam et al., 2000; DelVecchio et al., 2002; Haukness et al., 2002; Murakami et al., 2002; Schmitz et al., 2003; Ekelund et al., 2005; Bianco et al., 2006; Rivera et al., 2006; Luca-Harari et al., 2008; Maripuu et al., 2008; Chan et al., 2009; Hraoui et al., 2011; Kittang et al., 2011; Michaelsen et al., 2011; Strus et al., 2017; Bencardino et al., 2019; Yu et al., 2021; Li et al., 2022)
*speB*	4		268/280	583/632	0.60	0.11;3.26	57.4	(Schmitz et al., 2003; Luca-Harari et al., 2008; Strus et al., 2017; Yu et al., 2021)
*speC*	19		606/1,159	1,781/2,841	0.89	0.63;1.27	71.5	(Tyler et al., 1992; Hsueh et al., 1998; Descheemaeker et al., 2000; DelVecchio et al., 2002; Haukness et al., 2002; Murakami et al., 2002; Schmitz et al., 2003; Ekelund et al., 2005; Rivera et al., 2006; Luca-Harari et al., 2008; Maripuu et al., 2008; Chan et al., 2009; Hraoui et al., 2011; Kittang et al., 2011; Michaelsen et al., 2011; Strus et al., 2017; Bencardino et al., 2019; Yu et al., 2021; Li et al., 2022)
*speF*	4		236/256	373/390	0.97	0.42;2.22	0	(Hsueh et al., 1998; Schmitz et al., 2003; Chan et al., 2009; Yu et al., 2021)
*speG*	8		517/559	1,189/1,480	1.80	1.69;4.71	78.7	(Murakami et al., 2002; Schmitz et al., 2003; Rivera et al., 2006; Kittang et al., 2011; Michaelsen et al., 2011; Strus et al., 2017; Yu et al., 2021; Li et al., 2022)
*speH*	12		163/865	607/2,078	0.78	0.60;1.01	17.4	(Murakami et al., 2002; Schmitz et al., 2003; Ekelund et al., 2005; Rivera et al., 2006; Luca-Harari et al., 2008; Maripuu et al., 2008; Kittang et al., 2011; Michaelsen et al., 2011; Strus et al., 2017; Bencardino et al., 2019; Yu et al., 2021; Li et al., 2022)
*speI*	9		87/648	364/1,662	0.85	0.65;1.13	0	(Ekelund et al., 2005; Rivera et al., 2006; Maripuu et al., 2008; Kittang et al., 2011; Michaelsen et al., 2011; Strus et al., 2017; Bencardino et al., 2019; Yu et al., 2021; Li et al., 2022)
*speJ*	9		241/619	369/1,220	0.97	0.65;1.44	49	(Schmitz et al., 2003; Rivera et al., 2006; Maripuu et al., 2008; Kittang et al., 2011; Michaelsen et al., 2011; Strus et al., 2017; Coppens et al., 2019; Yu et al., 2021; Li et al., 2022)
*speK*	6		51/366	181/1,152	1.55	0.43;5.66	89.6	(Kittang et al., 2011; Strus et al., 2017; Bencardino et al., 2019; Coppens et al., 2019; Yu et al., 2021; Li et al., 2022)
*speL*	7		28/394	89/1,273	1.49	0.92;2.41	0	(Rivera et al., 2006; Kittang et al., 2011; Michaelsen et al., 2011; Strus et al., 2017; Bencardino et al., 2019; Yu et al., 2021; Li et al., 2022)
*speM*	8		63/448	133/1,310	**1.64**	1.06;2.52	24.7	(Rivera et al., 2006; Maripuu et al., 2008; Kittang et al., 2011; Michaelsen et al., 2011; Strus et al., 2017; Bencardino et al., 2019; Yu et al., 2021; Li et al., 2022)
*ssa*	15		241/1,014	928/2,585	1.05	0.61;1.83	81.3	(Descheemaeker et al., 2000; Murakami et al., 2002; Schmitz et al., 2003; Ekelund et al., 2005; Rivera et al., 2006; Luca-Harari et al., 2008; Maripuu et al., 2008; Chan et al., 2009; Hraoui et al., 2011; Kittang et al., 2011; Michaelsen et al., 2011; Strus et al., 2017; Bencardino et al., 2019; Yu et al., 2021; Li et al., 2022)
*smeZ*	10		446/651	1,071/1,430	1.02	0.48;2.17	80	(Schmitz et al., 2003; Rivera et al., 2006; Luca-Harari et al., 2008; Maripuu et al., 2008; Chan et al., 2009; Kittang et al., 2011; Strus et al., 2017; Bencardino et al., 2019; Yu et al., 2021; Li et al., 2022)
*prtf1*	3		29/55	200/369	**0.42**	0.20;0.87	0	(Bianco et al., 2006; Luca-Harari et al., 2008; Bencardino et al., 2019)
*pnga3*	1		74/236	265/417	**0.26**	0.19;0.37	–	(Li et al., 2022)
*sda1*	1		36/236	94/417	**0.62**	0.41;0.94	–	(Li et al., 2022)
*sic*	1		24/236	89/417	**0.42**	0.26;0.68	–	(Li et al., 2022)
*NADase 330G*	1		173/236	371/417	**0.34**	0.22;0.52	–	(Li et al., 2022)
*hasA*	1		163/236	225/417	**1.91**	1.36;2.67	–	(Li et al., 2022)

iGAS, invasive GAS infections; non-iGAS, non-invasive GAS infections; OR, odds ratio; CI, confidence interval; **bold typeface**, significant association.

As a subgroup, WGS-based studies rated as having a low risk of bias demonstrated significant associations of *hasA* [1 study, n = 653; OR, 1.91 (95%CI, 1.36; 2.67)] and *speG* [1 study, n = 653; OR, 2.83 (95%CI, 1.63; 4.92)] with iGAS infection. In contrast, significant inverse associations were observed for *speA* [1 study, n = 653; OR, 0.44 (95%CI, 0.27; 0.73)], *speK* [2 studies, n = 678; OR, 0.26 (95%CI, 0.15; 0.45)], *ssa* [1 study, n = 653; OR, 0.15 (95%CI, 0.08; 0.26)], *smeZ* [1 study, n = 653; OR, 0.42 (95%CI, 0.29; 0.61)], *NaDase 330G* [1 study, n = 653; OR, 0.34 (95%CI, 0.22; 0.52)], *sic* [1 study, n = 653; OR, 0.42 (95%CI, 0.26; 0.68)], *sda1* [1 study, n = 653; OR, 0.62 (95%CI, 0.41; 0.94)], and *pnga3* [1 study, n = 653; OR, 0.26 (95%CI, 0.19; 0.37)] with invasive GAS infections (**Table 3**). No heterogeneity was observed in the studies comprising the meta-analysis.

**Table 3:** Study data used in meta-analyses of virulence factors and invasive infection (lab method: WGS, low ROB).

Virulence factor	No. of studies	No. of iGAS	No. of non-iGAS	Pooled OR	95%CI	Heterogeneity (I^2^) %	Studies used (reference no.)
*speA*	1	22/236	79/417	**0.44**	0.27;0.73	–	(Li et al., 2022)
*speC*	1	169/236	302/417	0.96	0.67;1.37	–	(Li et al., 2022)
*speG*	1	219/236	342/417	**2.83**	1.63;4.92	–	(Li et al., 2022)
*speH*	1	36/236	78/417	0.78	0.51;1.21	–	(Li et al., 2022)
*speI*	1	35/236	72/417	0.83	0.54;1.30	–	(Li et al., 2022)
*speJ*	2	85/259	95/419	1.28	0.89;1.85	0	(Coppens et al., 2019; Li et al., 2022)
*speK*	2	20/259	98/419	**0.26**	0.15;0.45	0	(Coppens et al., 2019; Li et al., 2022)
*speL*	1	18/236	17/417	1.94	0.98;3.85	–	(Li et al., 2022)
*speM*	1	18/236	17/417	1.94	0.98;3.85	–	(Li et al., 2022)
*ssa*	1	15/236	132/417	**0.15**	0.08;0.26	–	(Li et al., 2022)
*smeZ*	1	162/236	350/417	**0.42**	0.29;0.61	–	(Li et al., 2022)
*NaDase 330G*	1	173/236	371/417	**0.34**	0.22;0.52	–	(Li et al., 2022)
*hasA*	1	163/236	225/417	**1.91**	1.36;2.67	–	(Li et al., 2022)
*sic*	1	24/236	89/417	**0.42**	0.26;0.68	–	(Li et al., 2022)
*sda1*	1	36/236	94/417	**0.62**	0.41;0.94	–	(Li et al., 2022)
*pnga3*	1	74/236	265/417	**0.26**	0.19;0.37	–	(Li et al., 2022)

iGAS, invasive GAS infections; non-iGAS, non-invasive GAS infections; OR, odds ratio; CI, confidence interval; **bold typeface**, significant association

Where reported, *emm types* (2,797 isolates) associated with invasive infection represented 27 different *emm clusters* (out of a possible 48) (**Supplementary Table S7**—available at https://doi.org/10.25375/uct.23708346) (Sanderson-Smith et al., 2014). Of these, six *emm clusters* were significantly associated with invasive GAS infection: AC3 [*emm1*; n = 25 studies; OR, 1.63 (95%CI, 1.44; 1.84], AC5 [*emm3*; n = 21 studies; OR, 2.15 (95%CI, 1.75; 2.64)], and E3 [*emm9*, *emm25*, *emm44*, *emm49*, *emm58*, *emm82*, *emm87*, *emm103*, *emm113*, *emm118*, *emm180*, *emm183*, and *emm219*; n = 16 studies; OR, 1.38 (95%CI, 1.13; 1.68)]. Clusters AC4 [*emm12*, *emm39*; n = 23 studies; OR, 0.40 (95%CI, 0.35; 0.47)], E1 [*emm4*, *emm60*, *emm78*, *emm165*, *emm176*; n = 22 studies; OR, 0.53 (95%CI, 0.45; 0.63)], and M6 [*emm6*; n = OR, 0.59 (95%CI, 0.45; 0.79)] were inversely associated with invasive GAS infection (**Table 4**).

**Table 4:** List of *emm clusters* significantly associated with invasive GAS infection.

emm cluster	emm type	Number of studies	Total number of isolates	Odds of association	95% confidence interval
AC3	*emm1*	25	7,853	1.63	1.44;1.84
AC4	*emm12*, *emm39*	23	7,786	0.40	0.35;0.47
AC5	*emm3*	21	6,064	2.15	1.75;2.64
E1	*emm4*, *emm60*, *emm78*, *emm165*, *emm176*	22	10,855	0.53	0.45;0.63
E3	*emm9*, *emm25*, *emm44*, *emm49*, *emm58*, *emm82*, *emm87*, *emm103*, *emm113*, *emm118*, *emm180*, *emm183*, *emm219*	16	22,965	1.38	1.13;1.68
M6	*emm6*	18	5,399	0.59	0.45;0.79

More than 97% of the cluster-associated isolates belonged to 11 *emm clusters* (in decreasing order of frequency: E4, AC3, E6, E3, E1, AC4, AC5, E2, M6, D4, M5) (**Supplementary Table S7**—available at https://doi.org/10.25375/uct.23708346). A total of 20 clusters (n = 4,099 isolates) were documented among the isolates associated with the significant virulence factors (*speA*, *speG*, *speK*, *speM*, *ssa*, *smeZ*) (**Supplementary Table S8**—available at https://doi.org/10.25375/uct.23708346). Five clusters (AC4, E2, E3, E4, E6) were frequently seen with *speG* and *smeZ*; cluster E1 with *ssa* and *smeZ*; cluster AC3 with *speA*, *speG*, and *smeZ*; and cluster AC5 with *speA*, *speG*, *ssa*, and *smeZ*. Clusters D4 and M6 were frequently seen with *smeZ*, and cluster M18 with *speA* (**Supplementary Table S9**—available at https://doi.org/10.25375/uct.23708346).

## Discussion

This review comprising a global investigation of virulence factors in invasive and non-invasive GAS infection provides reliable evidence for the association of GAS genetic elements with invasive disease. We identified 45 GAS putative virulence factors across 32 studies in our systematic review. Meta-analysis of high-quality studies identified a significant association, correlating positively and inversely, between genes and invasive disease. Below is the synthesis of virulence factors determined to be significantly associated with invasive disease as assembled through our review.

Among the chromosomally encoded superantigens, a lack of association between *smeZ* and invasive GAS disease was observed in this review, which is in agreement with an earlier study (Rogers et al., 2007). In this review, a significant association of *speG* and invasive GAS disease was seen. *SpeG* has been implicated in modulating host inflammatory responses and inhibiting complement activation (Friães et al., 2012). However, when considering WGS studies only, no associations were seen between *speG* and invasive disease, correlating with reports elsewhere (Proft and Fraser, 2003; Proft et al., 2003). Furthermore, *speG* has been reported in both invasive and non-invasive GAS, suggesting that virulence may, instead, be mediated by other elements in invasive GAS disease.

*SpeA*, *speK*, and *speM* are phage-encoded superantigens, mainly acquired via horizontal gene transfer; their differences result from the loss or acquisition of prophages. *SpeA* has been shown to promote bacterial adhesion and may play a role in invasion and dissemination of the bacterium. Most of the strains associated with severe streptococcal infections have been shown to produce the SpeA toxin (Yu and Ferretti, 1989; Hauser et al., 1991). In this review, we found that *speA* was associated with invasive GAS infection among PCR-based studies, which is commensurate with reports elsewhere (Hauser et al., 1991; Musser et al., 1991). However, when referring to the WGS studies, we also observed a significant inverse association of *speA* with invasive GAS infection. *SpeK* is a pseudogene characterized by an incomplete open reading frame (ORF) (Ferretti et al., 2001). Individuals infected with the M3 GAS strain MGAS315, containing phage genes, exhibited antibodies against *speK*, suggesting that this protein is produced *in vivo* (Beres et al., 2002). Our findings showed that *speK* had a significant association with invasive GAS among PCR-based studies. However, in WGS studies only, we observed a significant inverse association of *speK* with invasive GAS infection, contrasting with the PCR-based results. Our findings revealed that *speM* was significantly associated with invasive GAS infection.

Streptococcal superantigen (*ssa*) has been described in M3-related toxic shock syndrome isolates (Mollick et al., 1993), suggesting it to be a potential GAS virulence factor. In this review, we observed a significant inverse association with *ssa* and invasive GAS infection. *Sda1* encodes for an extracellular nuclease that displays a strong sequence non-specific nuclease activity on DNA substrates and is thought to play a role in evasion of the host’s innate immune response by degradation of the DNA component of neutrophil extracellular traps (NETs) and macrophage extracellular traps (Uchiyama et al., 2012). Although earlier studies using a murine model indicated the significance of *sda1* in enhancing GAS virulence during necrotizing fasciitis (Buchanan et al., 2006), our findings reveal an inverse correlation between *sda1* and invasive disease. This implies that virulence may be influenced by factors beyond *sda1*, considering the critical role of extracellular DNA-degrading activity in GAS invasive disease virulence (Uchiyama et al., 2012).

Hyaluronan synthase (*hasA*) increases virulence by aiding in evasion of the host immune system (Dougherty and Van de Rijn, 1994; Wessels, 2019). *HasA* plays an important role in invasive GAS disease (Ashbaugh et al., 1998). This is highlighted by the enhanced production of capsules by the invasive disease-associated serotype M3 isolates relative to other isolates and by the *in vivo* selection of isolates during invasive diseases that show enhanced capsule production (Shea et al., 2011). This review found that *hasA* is significantly associated with invasive infection in WGS-based studies. Unfortunately, the included study in our review looked at isolates from a single location and at a single time; two of the three most highly represented serotypes are M89 and M4, which are known to partially (M89, certain clades) or fully (M4) lack the *has* operon and are not in the top three M types listed in the study overall (Li et al., 2022) (**Supplementary Figure S1**).

The enzymatic activity of NAD+–glycohydrolase (NADase) is essential in GAS virulence; NADase works interdependently with streptolysin O (SLO), a pore-forming toxin, to facilitate pore formation during GAS infection (Mozola and Caparon, 2015). Despite several clinical GAS isolates being deficient in NADase activity, they may still exhibit cytotoxicity comparable to that of NADase-proficient strains (Riddle et al., 2010; Chandrasekaran et al., 2013). NADaseG330D, a frequently occurring genetic variation characterized by the presence of aspartate at position 330 of NADase, exhibits a lack of observable NADase activity (Chandrasekaran et al., 2013). Nevertheless, NADaseG330D remains a potent virulence factor and demonstrates the ability to interact with SLO in a manner similar to that of the wild-type NADase (Velarde et al., 2017). In this study, however, NADaseG330D was inversely associated with invasive GAS infection.

This review showed that PrtF1 was inversely associated with invasive GAS infection in PCR-based studies. Protein F1 (PrtF1/sfb1) is a fibronectin-binding protein, reported to promote epithelial cell adhesion and internalization. Hyland et al. demonstrated that PrtF1 expression elicits increased invasion of epithelial cells and resistance to phagocytosis, when expressed in M1 *Streptococcus pyogenes* strains (Hyland et al., 2007).

Westman et al. illustrated that streptococcal inhibitor of the complement (*sic*) is associated with invasive infection; *sic* is a secreted virulence factor that confers protection to GAS and performs multifunctional activities such as interfering with complement function and binding to various ligands essential for host colonization (Fernie-King et al., 2004; Pence et al., 2010; Frick et al., 2011; Westman et al., 2018). The contrast of the findings in this review showing an inverse association of *sic* with invasive disease may be due to Westman et al. only focusing on specific serotypes in iGAS, thus suggesting that virulence factors other than *sic* mediate invasive infection.

A single study by Chochua et al. found that *pnga3* was present in 55.6% of 1,454 invasive GAS isolates. *Pnga3* is a clade 3 upregulated promoter of the nga operon that encodes NADase and streptolysin O (Chochua et al., 2017). In this review, one study documented *pnga3* to be inversely associated with invasive GAS infection as compared with non-invasive isolates. However, data on the association of *pnga3* and clinical phenotypes are relatively scarce, thus requiring more studies to correlate these findings.

This review found 12 *emm types* significantly associated with invasive GAS infection. Similar patterns of *emm types* causing invasive disease were observed in other studies (O’Brien et al., 2002; Sharkawy et al., 2002; Naseer et al., 2016). Utilizing the cluster classification of the numerous *emm types*, 11 prevalent *emm clusters* were observed in invasive GAS isolates: clusters AC3, AC5, and E3 were found to be significantly associated with invasive GAS infection. Our findings correlate, albeit in a different order, with previous studies describing these clusters and their corresponding *emm types* in invasive infection (Smeesters et al., 2017; Friães et al., 2019; Jabang et al., 2021; Zangarini et al., 2023). We observed a close relationship between *emm cluster* and the significant virulence-associated factors, which correlates with results from China (Lu et al., 2017). More than 70% of isolates from the major cluster A-C3 (*emm1*) harbored *speA*, *speG*, and *smeZ*, correlating with a study performed by Gergova et al. (2019). The link between *emm type/cluster* and occurrence of virulence factors may be greatly conserved for the most virulent *emm types*, rendering them more pathogenic (Vlaminckx et al., 2003).

Collectively, our data contribute to an understanding of the interrelational nature of *emm type/clusters* and other virulence determinants in streptococcal pathogenesis and clinical outcomes. The vast amount of functional redundancy among superantigens emphasizes the biological significance of these elements and also suggests that host factors have a substantial contribution in the outcome of GAS infection.

One of the strengths of this review is the use of multiple databases and a broad inclusive search strategy so as to prevent overlooking eligible articles. Quality was assured through the inclusion, only, of articles of high quality, thus allowing for comparisons across the studies. Challenges in conducting this review arose from unclear definitions of invasive disease, requiring assumptions on the part of the reviewer (based on the isolation site reported) as well as variation in methods used to identify the virulence factors in this review.

We acknowledge the limitation of the PCR method employed in earlier association studies, which may have been confounded by allelic variants not as yet detected (Commons et al., 2014); thus, the range of primer sequences may not have been optimal to identify several allelic variants of the single superantigen. In comparison, WGS methods offer comprehensive detection capabilities as they can identify sequences rapidly and accurately without the need for prior knowledge of specific targets. Secondly, we acknowledge that low numbers of isolates included in studies may impact meta-analyses; thus, we conducted subgroup analysis according to molecular method. Unfortunately, not having individual data precluded us from studying a potential combined effect of virulence factors.

In conclusion, we acknowledge that the limitation of only focusing on gene data has implications in interpreting these results; it must be borne in mind that the PCR and WGS methods do not confirm function, especially if single‐nucleotide polymorphisms (SNPs) or insertions and deletions (INDELs) are present, given that uncharacterized SNPs may negate function. Nevertheless, this systematic review provides the latest data on the association of virulence factors with iGAS, presenting evidence for a possible relationship between the *hasA* gene and invasive infection. Also, we document an inverse association of *smeZ*, *sic*, *sda1*, and *pnga3* genes with iGAS; this inverse association with invasive infection may be due to the presence of unknown virulence genes in GAS lineages. There is mixed evidence regarding the association of *SpeA*, *speK*, and *speG* with GAS virulence; thus, it is unclear if they are markers of invasive infection. The occurrence of specific genes encoding these virulence factors will serve to inform further research addressing the role of GAS virulence factors in both invasive and non-invasive GAS infections.

## Author contributions

KR: Conceptualization, Data curation, Formal Analysis, Investigation, Methodology, Software, Validation, Visualization, Writing – original draft, Writing – review & editing. MS: Data curation, Methodology, Validation, Writing – review & editing. KE: Data curation, Validation, Writing – review & editing. CM: Supervision, Validation, Writing – review & editing. LZ: Funding acquisition, Investigation, Resources, Writing – review & editing. ME: Conceptualization, Formal Analysis, Methodology, Software, Validation, Visualization, Writing – review & editing

## Supplementary material

Engel, Mark; Rampersadh, Kimona (2023). Supplementary Material - Group A streptococcus (GAS) virulence factors associated with invasive disease: a systematic review. University of Cape Town. Journal contribution. https://doi.org/10.25375/uct.23708346.v4

Supplementary Figure 1 The most prevalent *emm types* identified amongst GAS studies.

Supplementary Table 1 Search strategy for PubMed database.

Supplementary Table 2 Characteristics of excluded studies.

Supplementary Table 3 Risk of bias of the included studies.

Supplementary Table 4 List of *emm types* significantly associated with invasive GAS infection.

Supplementary Table 5 Study data of all virulence factors in all the studies (classification-cell surface, secretory, both or other).

Supplementary Table 6 Meta-analyses of the association of secretory virulence factors and invasive infection (lab method: PCR; Low ROB).

Supplementary Table 7 Distribution of *emm cluster* in invasive GAS infections.

Supplementary Table 8 Extract of the more significant *emm clusters* associated with the significant virulence factors.

Supplementary Table 9 Summary of *emm clusters* (>2 studies) associated with the significant virulence factors.
