# Supplementary material for: Presence of Group A streptococcus frequently assayed virulence genes in invasive disease: a systematic review and meta-analysis
Source: Front Cell Infect Microbiol. 2024 Jun 3;14:1337861. doi: 10.3389/fcimb.2024.1337861 (PMC11270091; doi:10.3389/fcimb.2024.1337861)
Supplement: Supplementary Figure 1 — The most prevalent emm types identified amongst GAS studies. [file DataSheet_1.pdf]

Supplementary data

Fig S1. The most prevalent emm types identified amongst GAS studies.

Table S1. Search strategy for PubMed database.

Table S2. Characteristics of excluded studies.

Table S3. Risk of bias of the included studies.

Table S4. List of emm types significantly associated with invasive GAS infection.

Table S5. Study data of all virulence factors in all the studies (classification-cell surface, secretory, both or other).

Table S6. Meta-analyses of the association of secretory virulence factors and invasive infection (lab method: PCR; Low ROB).

Table S7. Distribution of emm cluster in invasive GAS infections.

Table S8. Extract of the more significant emm clusters associated with the significant virulence factors.

Table S9. Summary of emm clusters (>2 studies) associated with the significant virulence factors.

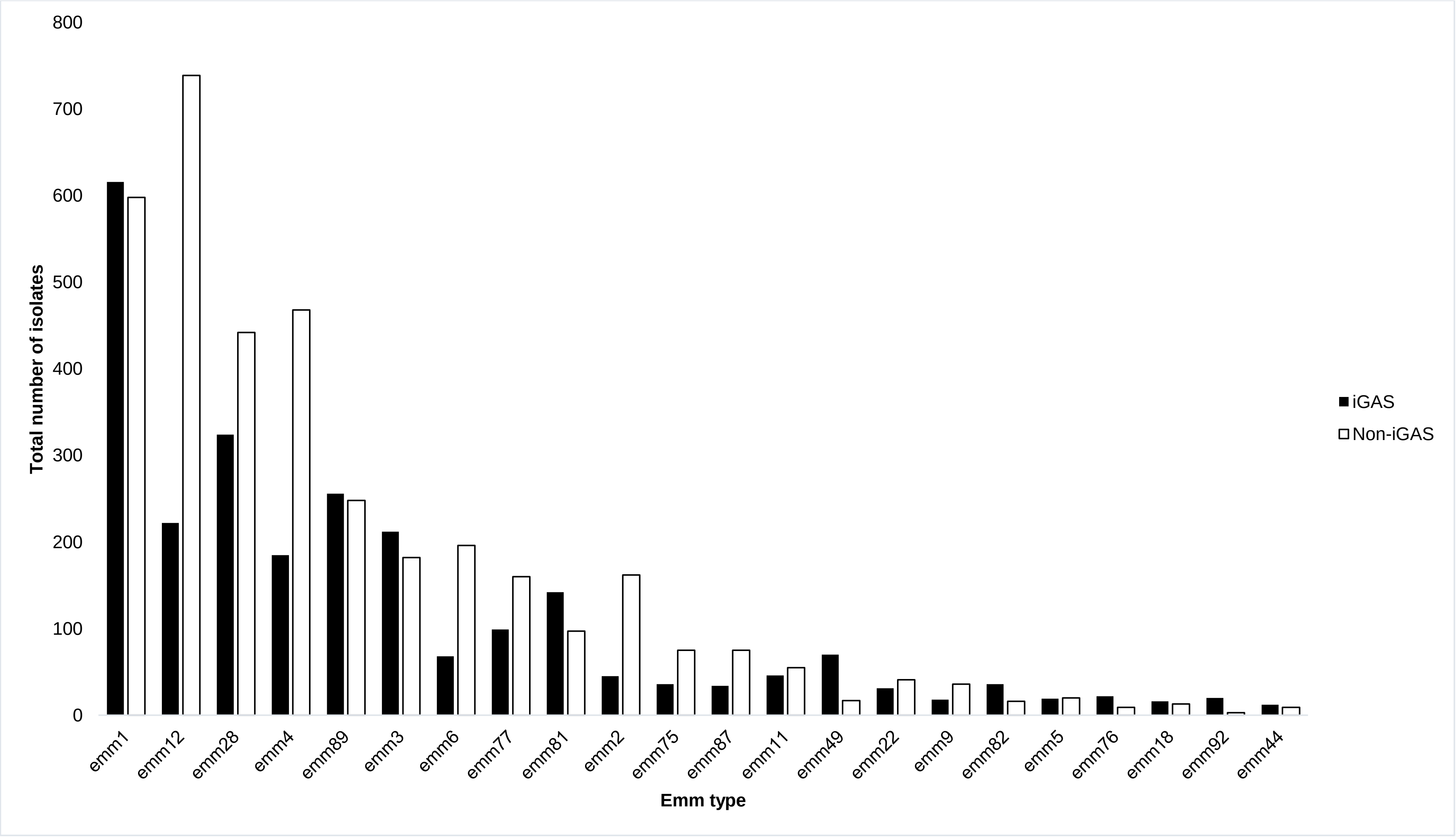

Fig S1. The most prevalent emm types identified amongst GAS studies.

Table S1. Search strategy for PubMed database.

Search number	Search Details
5	#3 AND #4
4	"Invasive GAS"[All Fields] OR "iGAS"[All Fields] OR "sepsis"[All Fields] OR "septicaemia"[All Fields] OR "Streptococcal toxic shock syndrome"[All Fields] OR "Necrotizing fasciitis"[All Fields] OR (("streptococcal infections"[MeSH Terms] OR ("streptococcal"[All Fields] AND "infections"[All Fields]) OR "streptococcal infections"[All Fields] OR "strep"[All Fields]) AND ("bacteraemia"[All Fields] OR "bacteremia"[MeSH Terms] OR "bacteremia"[All Fields] OR "bacteraemias"[All Fields] OR "bacteremias"[All Fields])) OR (("gas"[All Fields]) AND ("blood"[MeSH Subheading] OR "blood"[All Fields] OR "blood"[MeSH Terms] OR "bloods"[All Fields] OR "haematology"[All Fields] OR "hematology"[MeSH Terms] OR "hematology"[All Fields] OR "haematoma"[All Fields] OR "hematoma"[MeSH Terms] OR "hematoma"[All Fields] OR "haemorrhage"[All Fields] OR "hemorrhage"[MeSH Terms] OR "hemorrhage"[All Fields] OR "haemorrhages"[All Fields] OR "hemorrhages"[All Fields] OR "haemorrhagic"[All Fields] OR "haemorrhaging"[All Fields] OR "hematologies"[All Fields] OR "haematomas"[All Fields] OR "hematomas"[All Fields] OR "hematoma s"[All Fields] OR "hematomae"[All Fields] OR "hemorrhaged"[All Fields] OR "hemorrhagic"[All Fields] OR "hemorrhagical"[All Fields] OR "hemorrhaging"[All Fields]) AND ("infect"[All Fields] OR "infectability"[All Fields] OR "infectable"[All Fields] OR "infectant"[All Fields] OR "infectants"[All Fields] OR "infected"[All Fields] OR "infecteds"[All Fields] OR "infectibility"[All Fields] OR "infectible"[All Fields] OR "infecting"[All Fields] OR "infection s"[All Fields] OR "infections"[MeSH Terms] OR "infections"[All Fields] OR "infection"[All Fields] OR "infective"[All Fields] OR "infectiveness"[All Fields] OR "infectives"[All Fields] OR "infectivities"[All Fields] OR "infects"[All Fields] OR "pathogenicity"[MeSH Subheading] OR "pathogenicity"[All Fields] OR "infectivity"[All Fields])) OR (group a[Author] AND ("streptococcus"[MeSH Terms] OR "streptococcus"[All Fields] OR "streptococcal"[All Fields]) AND ("postpartum period"[MeSH Terms] OR ("postpartum"[All Fields] AND "period"[All Fields]) OR "postpartum period"[All Fields] OR "postpartum"[All Fields]) AND "metritis"[All Fields]) OR (("streptococcus"[MeSH Terms] OR "streptococcus"[All Fields] OR "streptococcal"[All Fields]) AND ("postpartum period"[MeSH Terms] OR ("postpartum"[All Fields] AND "period"[All Fields]) OR "postpartum period"[All Fields] OR "postpartum"[All Fields]) AND ("infect"[All Fields] OR "infectability"[All Fields] OR "infectable"[All Fields] OR "infectant"[All Fields] OR "infectants"[All Fields] OR "infected"[All Fields] OR "infecteds"[All Fields] OR "infectibility"[All Fields] OR "infectible"[All Fields] OR "infecting"[All Fields] OR "infection s"[All Fields] OR "infections"[MeSH Terms] OR "infections"[All Fields] OR "infection"[All Fields] OR "infective"[All Fields] OR "infectiveness"[All Fields] OR "infectives"[All Fields] OR "infectivities"[All Fields] OR "infects"[All Fields] OR "pathogenicity"[MeSH Subheading] OR "pathogenicity"[All Fields] OR "infectivity"[All Fields]))
3	#1 AND #2
2	"Genetic elements"[All Fields] OR "superantigen*"[All Fields] OR "prophage*"[All Fields] OR "virulence factors"[All Fields] OR "virulent factors"[All Fields] OR "genome components"[All Fields]
1	"Streptococcus pyogenes"[All Fields] OR "Group A Streptococcus"[All Fields] OR "GAS"[All Fields] OR "Strep A"[All Fields]

This search strategy was modified for Scopus and Web of Science databases.

Table S2. Characteristics of excluded studies based on full-text.

Author	Year	Title	Reason
Nasser (1)	2014	Evolutionary pathway to increased virulence and epidemic group A Streptococcus disease derived from 3,615 genome sequences	No comparison of iGAS/non-iGAS
Meisal (2)	2009	Molecular characteristics of pharyngeal and invasive emm3 Streptococcus pyogenes strains from Norway, 1988-2003	No comparison of iGAS/non-iGAS
Al-Shahib (3)	2016	Emergence of a novel lineage containing a prophage in emm/M3 group A Streptococcus associated with upsurge in invasive disease in the UK	No comparison of iGAS/non-iGAS
Vlaminckx (4)	2007	Dynamics in prophage content of invasive and non-invasive M1 and M28 Streptococcus pyogenes isolates in The Netherlands from 1959 to 1996	No comparison of iGAS/non-iGAS
Rogers (5)	2007	Strain prevalence, rather than innate virulence potential, is the major factor responsible for an increase in serious group A streptococcus infections	No comparison of iGAS/non-iGAS
Vikersfors (6)	2009	Severe group A streptococcal infections in Uppsala County, Sweden: clinical and molecular characterization of a case cluster from 2006 to 2007	No comparison of iGAS/non-iGAS
Akesson (7)	2004	Low antibody levels against cell wall-attached proteins of Streptococcus pyogenes predispose for severe invasive disease	Proteomic study
Azuma (8)	2004	Detection of circulating superantigens in an intensive care unit population	Proteomic study
Basma (9)	1999	Risk factors in the pathogenesis of invasive group A streptococcal infections: role of protective humoral immunity	Proteomic study
Yang (10)	2006	Variations in the protective immune response against streptococcal superantigens in populations of different ethnicity	Proteomic study
Arslan (11)	2013	Distribution of emm genotypes and antibiotic susceptibility of Streptococcus pyogenes strains: analogy with the vaccine in development	No virulence factors investigated
Chalker (12)	2017	Genome analysis following a national increase in Scarlet Fever in England 2014	No virulence factors investigated
Leung (13)	2018	Group a streptococcus disease in hong kong children: An overview	No virulence factors investigated
O'Brien (14)	1997	The changing epidemiology of group A Streptococcus infections	No virulence factors investigated
Parks (15)	2020	Elevated risk of invasive group A streptococcal disease and host genetic variation in the human leucocyte antigen locus	No virulence factors investigated
Sanson (16)	2019	Unexpected relationships between frequency of antimicrobial resistance, disease phenotype and emm type in group A Streptococcus	No virulence factors investigated
Kawabata (17)	2005	Virulence factors in Streptococcus pyogenes infection	Full text not available
Newton (18)	1997	Novel superantigens from streptococcal toxic shock syndrome Streptococcus pyogenes isolates	Full text not available
Brown (19)	2004	The Molecular Basis of Streptococcal Toxic Shock Syndrome	Wrong publication type
Cherneski (20)	2001	Necrotizing fasciitis	Wrong publication type
Cole (21)	2010	Molecular insight into invasive group A streptococcal disease	Wrong publication type
Steer (22)	2012	Invasive group a streptococcal disease: epidemiology, pathogenesis and management	Wrong publication type
Vlaminckx (23)	2007	Invasive Lancefield group A streptococcal infections in the Netherlands	Wrong publication type
Matsumoto (24)	2016	Description of the Pathogenic Features of Streptococcus pyogenes Isolates from Invasive and Non-Invasive Diseases in Aichi, Japan	Wrong publication type
Cleary (25)	1992	Clonal basis for resurgence of serious Streptococcus pyogenes disease in the 1980s	RFLP Study
Friães (26)	2012	Group A streptococci clones associated with invasive infections and pharyngitis in Portugal present differences in emm types, superantigen gene content and antimicrobial resistance	No breakdown of SAg in iGAS/non-iGAS

Author	Year	Title	Reason
Bogiel	2022	Assessment of the Relationship between Clinical Manifestation and Pathogenic Potential of Streptococcus pyogenes Strains-Distribution of Genes and Genotypes of Toxins	No breakdown of SAg in iGAS/non-iGAS

Table S3. Risk of bias of the included studies.

Study ID	RoB 1	RoB 2	RoB 3	RoB 4	RoB 5	RoB 6	ROB score	ROB
Bencardino 2019	1	1	1	1	1	0	5	Low
Bianco 2006	1	1	1	1	1	0	5	Low
Chan 2009	1	0	1	1	1	1	5	Low
Coppens 2019	1	0	1	1	1	1	5	Low
Creti 2005	1	1	0	1	1	0	4	Moderate
Darenberg 2007	1	0	1	1	1	0	4	Moderate
Descheemaeker 2000	1	1	1	1	1	0	5	Low
DelVecchio 2002	1	1	1	1	1	0	5	Low
Ekelund 2005	1	1	1	1	1	1	6	Low
Golińska 2016	0	1	1	1	1	0	4	Moderate
Haukness 2002	1	1	1	1	1	0	5	Low
Hraoui 2011	1	1	1	1	1	0	5	Low
Hsueh 1998	1	1	1	1	1	0	5	Low
Jing 2006	1	0	1	1	1	0	4	Moderate
Khan 2020	1	0	0	1	1	0	3	Moderate
Kittang 2011	1	1	1	1	1	1	6	Low
Li 2022	1	1	1	1	1	1	6	Low
Lintges 2010	1	0	1	1	1	0	4	Moderate
Luca-Harari 2008	1	0	1	1	1	1	5	Low
Maripuu 2008	1	1	1	1	1	0	5	Low
Meehan 2018	1	0	1	1	1	0	4	Moderate
Michaelsen 2011	1	1	1	1	1	0	5	Low
Muhtarova 2017	1	U	1	1	1	0	4	Moderate
Murakami 2002	1	1	1	1	1	0	5	Low
Mylvaganam 2000	1	1	1	1	1	0	5	Low
Nandi 2002	1	1	0	1	1	0	4	Moderate
Plainvert 2014	1	1	1	1	1	0	5	Low
Rivera 2006	1	1	1	1	1	1	6	Low
Schmitz 2003	1	1	1	1	1	0	5	Low
Strus 2017	1	1	1	1	1	0	5	Low
Tyler 1992	1	1	0	1	1	1	5	Low
Yu 2021	1	1	0	1	1	1	5	Low

1: study met the criteria; 0: the study did not meet the criteria; U: unclear.
ROB=Risk of bias; White=Low ROB; Grey=moderate ROB; Dark Grey=High ROB
Quality score: 1-2 high risk of bias; 3-4 moderate risk of bias; 5-6 low risk of bias
ROB Categories: 1-2, Representativeness of population; 3, Case definitions; 4, Study instrument reliability; 5, Data collection; 6, Limitations.

Table S4. List of emm types significantly associated with invasive GAS infection.

emm type	Odds of association	95% Confidence interval
emm1	1.63	1.44;1.84
emm12	0.41	0.35;0.48
emm3	2.15	1.75;2.64
emm83	4.47	1.30;15.37
emm4	0.61	0.51;0.73
emm27	9.46	1.21;74.13
emm76	3.33	1.53;7.26
emm90	8.52	1.06;68.36
emm92	6.41	1.90;21.67
emm104	0.21	0.06;0.75
emm49	6.25	3.66;10.67
emm82	3.04	1.67;5.51
emm2	0.48	0.34;0.66
emm77	0.75	0.58;0.97
emm89	1.60	1.33;1.92
emm11	1.50	1.01;2.24
emm81	1.46	1.12;1.90
emm6	0.59	0.45;0.79

Table S5. Study data of all virulence factors in all the studies (classification-cell surface, secretory, both or other).

Bacterium location	Virulence factor	Studies (n)	GAS isolates (n)	GAS isolates (N)	iGAS (n)	non-iGAS (n)	Studies used (Reference No.)
Cell surface	*prtF1*	3	229	424	29/55	200/369	(27-29)
	prtF2	1	73	122	0/5	73/117	(27)
	hasA	1	388	653	163/236	225/417	(30)
	sda1	1	130	653	36/236	94/417	(30)
	spy1325	1	34	67	13/30	21/37	(31)
	sla	4	49	331	11/37	38/294	(27, 31-33)
	srtB	1	60	122	1/5	59/117	(27)
	cpa	1	62	122	0/5	62/117	(27)
	bridge(region)	1	52	122	0/5	52/117	(27)
	fctA	1	40	122	0/5	40/117	(27)
	fctB	1	69	122	0/5	69/117	(27)
Secretory	*speA*	30	1629	4984	640/1543	989/3441	(27-56)
	speB	18	2127	2138	569/574	1558/1564	(27, 28, 31, 32, 34, 35, 38, 42, 44-47, 51-56)
	speC	27	2745	4991	729/1605	2016/3386	(27, 28, 30-34, 36-51, 54-57)
	speF	12	1674	1839	423/464	1251/1375	(28, 29, 31, 34, 38, 42, 45-47, 53, 55, 56)
	speG	15	2605	3340	972/1139	1633/2201	(30, 31, 33, 35, 36, 39-43, 45, 46, 48, 56, 57)
	speH	17	1022	3601	279/1191	743/2410	(27, 28, 30, 31, 33, 36, 37, 39-43, 45, 46, 48, 56, 57)
	speI	13	823	2968	215/974	608/1994	(27, 30, 31, 33, 37, 39-43, 45, 46, 57)
	speJ	14	1162	3024	461/1122	701/1902	(30, 31, 33, 35, 36, 39-43, 45, 46, 56, 57)
	speK	10	708	2703	215/869	493/1834	(27, 30, 31, 33, 35, 40, 42, 45, 46, 57)
	speL	11	438	2877	141/920	297/1957	(27, 30, 31, 33, 40-43, 45, 46, 57)
	speM	12	503	2968	167/974	336/1994	(27, 30, 31, 33, 39-43, 45, 46, 57)
	ssa	23	1588	4459	388/1413	1200/3046	(27, 28, 30-34, 36-48, 52, 56, 57)
	smeZ	17	2685	3377	908/1187	1777/2190	(27, 28, 30-36, 39, 40, 42, 43, 45, 46, 56, 57)
	sil	2	205	659	152/445	53/214	(32, 58)
	slo	3	459	459	66/66	393/393	(32, 42, 54)
	sof	1	82	120	26/45	56/75	(28)
	NaDase 330G	1	544	653	173/236	371/417	(30)

Bacterium location	Virulence factor	Studies (n)	GAS isolates (n)	GAS isolates (N)	iGAS (n)	non-iGAS (n)	Studies used (Reference No.)
	NaDase	1	64	114	34/63	30/51	(50)
	sic	1	113	653	24/236	89/471	(30)
	mac	1	204	238	29/35	175/203	(42)
	per	1	48	67	22/30	26/37	(31)
	Phage associated HNH homing endonuclease	1	21	25	19/23	2/2	(35)
	sdn	1	35	122	1/5	34/117	(27)
	sipA2	1	73	122	1/5	72/117	(27)
	mf	1	316	316	17/17	299/299	(48)
Both	*spyCEP*	1	238	238	35/35	203/203	(42)
	sdaB	1	238	238	35/35	203/203	(42)
	sdaD	1	132	238	20/35	112/203	(42)
	sdc	1	156	238	24/35	132/203	(42)
	spd3	1	123	238	19/35	104/203	(42)
Other	*pnga3*	2	339	653	74/236	265/417	(30)
	srtC2	1	67	122	0/5	67/117	(27)
	rofA	1	98	122	5/5	93/117	(27)
	nra	1	24	122	0/5	24/117	(27)

i-GAS= invasive GAS infections, non-iGAS= non-invasive GAS infections.

Table S6. Meta-analyses of the association of virulence factors and invasive infection (lab method: PCR; Low ROB).

Virulence factor	Studies (n)	iGAS (n)	non-iGAS (n)	Pooled OR	95% CI	Heterogeneity (I^2) (%)	Studies used (Reference No.)
speA	20	420/950	799/2621	**1.59**	1.10;2.30	64.4	(27-29, 33, 34, 37, 39, 41, 43-48, 50-52, 54-56)
speB	4	268/280	583/632	0.60	0.11;3.26	57.4	(28, 45, 46, 56)
speC	18	437/923	1479/2424	0.89	0.59;1.32	72.8	(27, 28, 33, 34, 37, 39, 43-48, 50, 51, 54-56)
speF	4	236/256	373/390	0.97	0.42;2.22	0	(34, 45, 55, 56)
speG	7	298/323	847/1063	1.70	0.49;5.89	79.5	(33, 41, 43, 45, 46, 48, 56)
speH	11	127/629	529/1661	0.79	0.57;1.09	24.7	(27, 28, 33, 37, 39, 41, 43, 45, 46, 48, 56)
speI	8	52/412	292/1245	0.86	0.59;1.25	3	(27, 33, 37, 39, 41, 43, 45, 46)
speJ	7	156/360	274/801	0.87	0.52;1.46	57.4	(33, 39, 41, 43, 45, 46, 56)
speK	4	31/107	83/733	**2.95**	1.81;4.80	0	(27, 33, 45, 46)
speL	6	10/158	72/856	1.14	0.57;2.26	0	(27, 33, 41, 43, 45, 46)
speM	7	45/212	116/893	1.52	0.88;2.64	34.1	(27, 33, 39, 41, 43, 45, 46)
ssa	14	226/778	796/2168	1.21	0.79;1.86	65.6	(27, 28, 33, 34, 37, 39, 41, 43-48, 56)
smeZ	9	284/415	721/1013	1.21	0.58;2.54	61.4	(27, 28, 33, 34, 39, 43, 45, 46, 56)
prtf1	3	29/55	200/369	**0.42**	0.20;0.87	0	(27-29)

i-GAS= invasive GAS infections, non-iGAS= non-invasive GAS infections. OR= Odds Ratio; CI= Confidence interval; **Bold Typeface** = Significant association

Table S7. Distribution of emm cluster in invasive GAS infections.

Emm cluster	Emm type	Total number of isolates for cluster	Cumulative %
E4	emm2, emm8, emm22, emm28, emm73, emm77, emm84, emm88, emm89, emm102, emm109, emm112, emm124	783	28.0%
AC3	emm1	616	50.0%
E6	emm11, emm42, emm59, emm63, emm65, emm67, emm75, emm81, emm85, emm94, emm158, emm172, emm182	260	59.3%
E3	emm9, emm25, emm44, emm49, emm58, emm82, emm87, emm103, emm113, emm118, emm180, emm183	202	66.5%
E1	emm4, emm60, emm78	204	73.8%
AC4	emm12, emm39	224	81.8%
AC5	emm3	212	89.4%
E2	emm27, emm50, emm66, emm68, emm76, emm90, emm92, emm104, emm106, emm110, emm117, emm168	89	92.6%
M6	emm6	68	95.0%
D4	emm33, emm41, emm43, emm53, emm64, emm80, emm83, emm86, emm91, emm93, emm101, emm108, emm116, emm119, emm192, emm230	50	96.8%
M5	emm5	19	97.5%
M18	emm18	16	98.1%
D2	emm32, emm71, emm100	10	98.4%
M29	emm29	9	98.7%
No emm cluster	emm62, emm69, emm147, emm187, 27G, stns554, stG1750	13	99.2%
M95	emm95	4	99.4%
M74	emm74	3	99.5%
D3	emm217	2	99.5%
M14	emm14	2	99.6%
M122	emm122	2	99.7%
M55	emm55	2	99.7%
AC1	emm142	1	99.8%
E5	emm174	1	99.8%
M236	emm236	1	99.9%
M17	emm17	1	99.9%
M23	emm23	1	99.9%
M24	emm24	1	100.0%
M179	emm179	1	100.0%

Table S8. Extract of the more significant emm clusters associated with the significant virulence factors.

Study ID	Emm type	Emm cluster	Total GAS isolates (N)	Number of GAS isolates (n)					
				speA	*speG*	*speK*	*speM*	*ssa*	*smeZ*
Ekeland 2005	emm1	AC3	100	82	N/A	N/A	N/A	11	N/A
Coppens 2019	emm1	AC3	25	25	25	3	N/A	N/A	25
Darenberg 2007	emm1	AC3	146	119	146	N/A	N/A	7	145
Jing 2006	emm1	AC3	25	21	N/A	N/A	N/A	10	19
Chan 2009	emm1	AC3	12	12	N/A	N/A	N/A	2	12
Luca-Harari 2008	emm1	AC3	37	35	N/A	N/A	N/A	2	36
Meehan 2018	emm1	AC3	188	187	184	4	0	1	188
Maripuu 2008	emm1	AC3	45	44	45	N/A	6	0	45
Michaelsen 2011	emm1	AC3	22	22	22	N/A	0	0	N/A
Rivera 2006	emm1	AC3	22	22	22	N/A	0	0	12
Bencardino 2019	emm1	AC3	18	9	N/A	1	1	2	2
Yu 2021	emm1	AC3	105	93	94	2	2	104	104
Murakami 2002	emm1	AC3	42	41	34	N/A	N/A	14	N/A
Creti 2005	emm1	AC3	30	27	N/A	N/A	N/A	N/A	N/A
Haukness 2002	emm1	AC3	13	13	N/A	N/A	N/A	N/A	N/A
Schmitz 2003	emm1	AC3	72	72	72	N/A	N/A	2	66
Kittang 2011	emm1	AC3	6	6	6	0	0	0	6
Ekeland 2005	emm12	AC4	69	8	N/A	N/A	N/A	13	N/A
Darenberg 2007	emm12	AC4	157	9	157	N/A	N/A	5	157
Jing 2006	emm12	AC4	20	7	N/A	N/A	N/A	8	17
Chan 2009	emm12	AC4	5	0	N/A	N/A	N/A	3	5
Luca-Harari 2008	emm12	AC4	26	0	N/A	N/A	N/A	0	25
Meehan 2018	emm12	AC4	92	8	86	1	1	23	90
Maripuu 2008	emm12	AC4	4	0	4	N/A	0	0	4
Michaelsen 2011	emm12	AC4	3	0	3	N/A	0	0	N/A
Rivera 2006	emm12	AC4	9	0	9	N/A	0	1	9
Bencardino 2019	emm12	AC4	11	0	N/A	4	2	1	1
Yu 2021	emm12	AC4	201	5	176	3	2	198	199
Murakami 2002	emm12	AC4	55	33	51	N/A	N/A	17	N/A

Study ID	Emm type	Emm cluster	Total GAS isolates (N)	Number of GAS isolates (n)					
				speA	*speG*	*speK*	*speM*	*ssa*	*smeZ*
Creti 2005	emm12	AC4	38	4	N/A	N/A	N/A	N/A	N/A
Haukness 2002	emm12	AC4	12	6	N/A	N/A	N/A	N/A	N/A
Schmitz 2003	emm12	AC4	23	4	21	N/A	N/A	2	0
Kittang 2011	emm12	AC4	22	0	22	0	0	4	22
Jing 2006	emm3	AC5	2	2	N/A	N/A	N/A	2	0
Luca-Harari 2008	emm3	AC5	2	2	N/A	N/A	N/A	1	1
Meehan 2018	emm3	AC5	148	138	147	64	2	100	146
Maripuu 2008	emm3	AC5	1	1	1	N/A	0	1	0
Michaelsen 2011	emm3	AC5	11	11	11	N/A	11	10	N/A
Rivera 2006	emm3	AC5	11	11	11	N/A	11	11	7
Yu 2021	emm3	AC5	2	2	0	2	2	0	2
Murakami 2002	emm3	AC5	11	11	6	N/A	N/A	11	N/A
Creti 2005	emm3	AC5	14	12	N/A	N/A	N/A	N/A	N/A
Haukness 2002	emm3	AC5	8	8	N/A	N/A	N/A	N/A	N/A
Schmitz 2003	emm3	AC5	51	51	51	N/A	N/A	45	1
Kittang 2011	emm3	AC5	11	11	11	11	0	11	11
Maripuu 2008	emm36	D1	1	0	1	N/A	0	1	1
Luca-Harari 2008	emm100	D2	1	0	N/A	N/A	N/A	1	1
Maripuu 2008	emm100	D2	1	0	1	N/A	1	0	1
Jing 2006	emm64	D4	1	0	N/A	N/A	N/A	0	1
Jing 2006	emm80	D4	4	4	N/A	N/A	N/A	0	4
Jing 2006	emm86	D4	1	0	N/A	N/A	N/A	0	1
Jing 2006	emm101	D4	3	1	N/A	N/A	N/A	0	3
Luca-Harari 2008	emm33	D4	1	0	N/A	N/A	N/A	1	1
Luca-Harari 2008	emm64	D4	1	0	N/A	N/A	N/A	0	1
Luca-Harari 2008	emm91	D4	1	0	N/A	N/A	N/A	0	1
Luca-Harari 2008	emm119	D4	1	0	N/A	N/A	N/A	0	1
Maripuu 2008	emm41.2	D4	1	0	1	N/A	0	0	1
Maripuu 2008	emm91	D4	1	0	1	N/A	0	0	1

Study ID	Emm type	Emm cluster	Total GAS isolates (N)	Number of GAS isolates (n)					
				speA	*speG*	*speK*	*speM*	*ssa*	*smeZ*
Maripuu 2008	emm93	D4	1	0	1	N/A	0	0	1
Rivera 2006	emm43.5	D4	1	0	1	N/A	0	0	1
Rivera 2006	emm64	D4	2	0	2	N/A	2	0	1
Rivera 2006	emm70	D4	1	0	1	N/A	1	0	1
Rivera 2006	emm83.4	D4	1	0	1	N/A	1	0	1
Creti 2005	emm80	D4	1	1	N/A	N/A	N/A	N/A	N/A
Ekeland 2005	emm4	E1	66	1	N/A	N/A	N/A	58	N/A
Darenberg 2007	emm4	E1	114	0	44	N/A	N/A	106	114
Jing 2006	emm4	E1	2	0	N/A	N/A	N/A	2	2
Chan 2009	emm4	E1	2	0	N/A	N/A	N/A	1	2
Luca-Harari 2008	emm4	E1	3	1	N/A	N/A	N/A	3	2
Luca-Harari 2008	emm78	E1	3	0	N/A	N/A	N/A	1	3
Meehan 2018	emm4	E1	110	2	27	1	2	95	108
Maripuu 2008	emm4	E1	2	0	2	N/A	0	2	2
Michaelsen 2011	emm4	E1	3	0	0	N/A	0	3	N/A
Rivera 2006	emm4	E1	11	1	0	N/A	0	10	6
Bencardino 2019	emm4	E1	3	3	N/A	0	2	10	8
Yu 2021	emm4.0	E1	8	2	2	2	2	6	7
Murakami 2002	emm4	E1	35	11	7	N/A	N/A	29	N/A
Haukness 2002	emm4	E1	7	6	N/A	N/A	N/A	N/A	N/A
Rivera 2006	emm165	E1	1	0	0	N/A	0	0	1
Kittang 2011	emm4	E1	9	0	1	0	0	8	9
Jing 2006	emm66	E2	2	0	N/A	N/A	N/A	0	2
Luca-Harari 2008	emm50/62	E2	1	0	N/A	N/A	N/A	0	1
Luca-Harari 2008	emm76	E2	9	0	N/A	N/A	N/A	0	9
Luca-Harari 2008	emm92	E2	2	0	N/A	N/A	N/A	2	2
Luca-Harari 2008	emm106	E2	3	0	N/A	N/A	N/A	2	2
Meehan 2018	emm90	E2	4	0	4	0	1	0	4
Maripuu 2008	emm66	E2	4	0	4	N/A	1	0	4

Study ID	Emm type	Emm cluster	Total GAS isolates (N)	Number of GAS isolates (n)					
				speA	*speG*	*speK*	*speM*	*ssa*	*smeZ*
Maripuu 2008	emm68	E2	1	0	1	N/A	0	0	4
Rivera 2006	emm50	E2	3	0	3	N/A	1	1	2
Murakami 2002	emm13	E2	17	14	17	N/A	N/A	2	N/A
Jing 2006	emm44	E3	1	1	N/A	N/A	N/A	0	0
Luca-Harari 2008	emm9	E3	2	0	N/A	N/A	N/A	0	2
Luca-Harari 2008	emm25	E3	1	0	N/A	N/A	N/A	0	1
Luca-Harari 2008	emm44/61	E3	2	0	N/A	N/A	N/A	0	2
Luca-Harari 2008	emm49	E3	7	6	N/A	N/A	N/A	0	2
Luca-Harari 2008	emm87	E3	1	1	N/A	N/A	N/A	1	0
Meehan 2018	emm9	E3	1	1	0	0	0	1	0
Meehan 2018	emm87	E3	16	0	16	0	2	14	16
Maripuu 2008	emm44	E3	1	0	1	N/A	0	0	0
Maripuu 2008	emm49	E3	1	0	1	N/A	0	0	0
Maripuu 2008	emm58	E3	1	0	1	N/A	0	0	1
Maripuu 2008	emm82	E3	1	0	1	N/A	0	0	1
Rivera 2006	emm9	E3	4	0	4	N/A	0	0	4
Rivera 2006	emm25.2	E3	4	0	4	N/A	0	4	3
Rivera 2006	emm44/61	E3	2	0	2	N/A	1	2	1
Rivera 2006	emm49.3	E3	1	1	1	N/A	0	0	1
Rivera 2006	emm58	E3	2	0	2	N/A	0	1	2
Rivera 2006	emm87	E3	3	0	3	N/A	0	3	2
Murakami 2002	emm58	E3	11	8	10	N/A	N/A	0	N/A
Murakami 2002	emm87	E3	6	3	5	N/A	N/A	2	N/A
Creti 2005	emm87	E3	8	1	N/A	N/A	N/A	N/A	N/A
Kittang 2011	emm82	E3	7	0	7	0	0	0	7
Kittang 2011	emm87	E3	14	0	14	0	0	13	13
Ekeland 2005	emm28	E4	83	12	N/A	N/A	N/A	12	N/A
Darenberg 2007	emm28	E4	229	6	228	N/A	N/A	6	228
Darenberg 2007	emm77	E4	91	0	27	N/A	N/A	5	90

Study ID	Emm type	Emm cluster	Total GAS isolates (N)	Number of GAS isolates (n)					
				speA	*speG*	*speK*	*speM*	*ssa*	*smeZ*
Darenberg 2007	emm89	E4	179	4	177	N/A	N/A	1	176
Jing 2006	emm8	E4	6	0	N/A	N/A	N/A	0	6
Jing 2006	emm28	E4	4	1	N/A	N/A	N/A	1	3
Jing 2006	emm77	E4	2	1	N/A	N/A	N/A	0	2
Jing 2006	emm88	E4	1	0	N/A	N/A	N/A	0	1
Chan 2009	emm22	E4	4	0	N/A	N/A	N/A	4	4
Chan 2009	emm73	E4	1	0	N/A	N/A	N/A	0	1
Chan 2009	emm112	E4	1	0	N/A	N/A	N/A	0	1
Luca-Harari 2008	emm2	E4	2	0	N/A	N/A	N/A	0	1
Luca-Harari 2008	emm8	E4	3	0	N/A	N/A	N/A	0	3
Luca-Harari 2008	emm22	E4	1	0	N/A	N/A	N/A	1	1
Luca-Harari 2008	emm28	E4	1	1	N/A	N/A	N/A	1	1
Luca-Harari 2008	emm77	E4	1	0	N/A	N/A	N/A	0	1
Luca-Harari 2008	emm102	E4	2	0	N/A	N/A	N/A	0	1
Meehan 2018	emm2	E4	2	0	0	0	0	0	1
Meehan 2018	emm22	E4	38	1	35	29	2	1	16
Meehan 2018	emm28	E4	106	8	105	40	0	3	105
Meehan 2018	emm77	E4	13	0	7	0	4	0	13
Meehan 2018	emm89	E4	84	1	77	4	0	3	83
Maripuu 2008	emm2	E4	4	1	4	N/A	1	0	2
Maripuu 2008	emm8	E4	4	0	4	N/A	4	0	4
Maripuu 2008	emm22	E4	1	0	1	N/A	0	0	1
Maripuu 2008	emm28	E4	6	0	6	N/A	0	0	6
Maripuu 2008	emm73	E4	1	1	1	N/A	0	0	1
Maripuu 2008	emm84	E4	1	0	1	N/A	0	0	0
Maripuu 2008	emm89	E4	2	0	2	N/A	0	0	2
Michaelsen 2011	emm28	E4	1	0	1	N/A	0	0	N/A
Rivera 2006	emm2	E4	4	0	4	N/A	4	0	0
Rivera 2006	emm22	E4	2	0	2	N/A	1	2	2

Study ID	Emm type	Emm cluster	Total GAS isolates (N)	Number of GAS isolates (n)					
				speA	*speG*	*speK*	*speM*	*ssa*	*smeZ*
Rivera 2006	emm28	E4	9	1	9	N/A	8	0	5
Rivera 2006	emm77	E4	8	0	1	N/A	0	1	8
Rivera 2006	emm89	E4	5	0	5	N/A	0	0	4
Bencardino 2019	emm89	E4	3	0	N/A	0	0	2	0
Yu 2021	emm2.0	E4	3	0	3	2	2	1	1
Yu 2021	emm22.0	E4	2	1	2	0	0	2	2
Yu 2021	emm28	E4	1	0	1	0	0	0	1
Yu 2021	emm89.0	E4	2	0	2	0	0	1	2
Murakami 2002	emm2	E4	12	3	10	N/A	N/A	9	N/A
Murakami 2002	emm28	E4	53	37	48	N/A	N/A	17	N/A
Murakami 2002	emm89	E4	8	5	7	N/A	N/A	0	N/A
Creti 2005	emm2	E4	8	5	N/A	N/A	N/A	N/A	N/A
Creti 2005	emm22	E4	13	1	N/A	N/A	N/A	N/A	N/A
Haukness 2002	emm2	E4	5	3	N/A	N/A	N/A	N/A	N/A
Haukness 2002	emm22	E4	3	2	N/A	N/A	N/A	N/A	N/A
Haukness 2002	emm28	E4	5	5	N/A	N/A	N/A	N/A	N/A
Haukness 2002	emm77	E4	4	4	N/A	N/A	N/A	N/A	N/A
Haukness 2002	emm89	E4	5	2	N/A	N/A	N/A	N/A	N/A
Schmitz 2003	emm28	E4	32	11	31	N/A	N/A	1	1
Kittang 2011	emm28	E4	27	3	27	14	0	0	27
Kittang 2011	emm89	E4	8	0	8	0	0	0	8
Darenberg 2007	emm81	E6	191	2	191	N/A	N/A	4	142
Jing 2006	emm63	E6	1	1	N/A	N/A	N/A	0	1
Jing 2006	emm75	E6	1	1	N/A	N/A	N/A	0	1
Jing 2006	emm94	E6	2	1	N/A	N/A	N/A	1	2
Luca-Harari 2008	emm65/69	E6	3	0	N/A	N/A	N/A	0	2
Luca-Harari 2008	emm75	E6	7	0	N/A	N/A	N/A	0	7
Luca-Harari 2008	emm81	E6	10	1	N/A	N/A	N/A	1	1
Meehan 2018	emm11	E6	17	0	17	0	0	1	17

Study ID	Emm type	Emm cluster	Total GAS isolates (N)	Number of GAS isolates (n)					
				speA	*speG*	*speK*	*speM*	*ssa*	*smeZ*
Meehan 2018	emm75	E6	23	2	23	20	20	2	22
Meehan 2018	emm81	E6	14	0	14	0	3	0	5
Maripuu 2008	emm75	E6	2	0	2	N/A	2	0	2
Maripuu 2008	emm81	E6	1	0	1	N/A	1	0	1
Maripuu 2008	emm85	E6	1	0	1	N/A	0	0	1
Rivera 2006	emm11	E6	8	0	8	N/A	0	0	5
Rivera 2006	emm59	E6	2	0	2	N/A	2	0	2
Rivera 2006	emm75	E6	3	0	3	N/A	3	0	3
Rivera 2006	emm81	E6	1	0	1	N/A	0	0	0
Yu 2021	emm75.0	E6	4	1	4	3	3	1	4
Murakami 2002	emm11	E6	11	9	11	N/A	N/A	2	N/A
Murakami 2002	emm75	E6	7	5	7	N/A	N/A	1	N/A
Haukness 2002	emm75	E6	2	1	N/A	N/A	N/A	N/A	N/A
Ekeland 2005	emm6	M6	57	3	N/A	N/A	N/A	10	N/A
Jing 2006	emm6	M6	1	1	N/A	N/A	N/A	1	0
Jing 2006	emm18	M18	5	3	N/A	N/A	N/A	1	5
Jing 2006	emm23	M23	1	0	N/A	N/A	N/A	1	1
Luca-Harari 2008	emm5	M5	1	0	N/A	N/A	N/A	0	1
Luca-Harari 2008	emm23	M23	1	1	N/A	N/A	N/A	1	1
Luca-Harari 2008	emm74	M74	1	0	N/A	N/A	N/A	0	1
Meehan 2018	emm5	M5	6	6	6	0	0	0	5
Maripuu 2008	emm14	M14	1	1	1	N/A	0	1	1
Maripuu 2008	emm19	M19	1	0	1	N/A	0	0	1
Rivera 2006	emm18	M18	1	1	1	N/A	1	0	1
Rivera 2006	emm29.2	M29	1	0	1	N/A	1	0	1
Bencardino 2019	emm29	M29	11	0	N/A	0	4	1	3
Murakami 2002	emm18	M18	8	7	4	N/A	N/A	2	N/A
Haukness 2002	emm5	M5	6	3	N/A	N/A	N/A	N/A	N/A
Haukness 2002	emm18	M18	5	2	N/A	N/A	N/A	N/A	N/A

Study ID	Emm type	Emm cluster	Total GAS isolates (N)	Number of GAS isolates (n)					
				speA	***speG***	***speK***	***speM***	***ssa***	***smeZ***
Luca-Harari 2008	emm95	M95	6	0	N/A	N/A	N/A	0	5
Luca-Harari 2008	emm6	M6	3	0	N/A	N/A	N/A	0	2
Meehan 2018	emm6	M6	58	9	53	42	0	0	58
Maripuu 2008	emm6	M6	1	1	1	N/A	1	0	1
Michaelsen 2011	emm6	M6	3	3	3	N/A	3	0	N/A
Rivera 2006	emm6	M6	3	3	3	N/A	3	0	3
Bencardino 2019	emm6	M6	12	3	N/A	7	0	0	4
Yu 2021	emm6	M6	14	12	9	12	12	4	14
Murakami 2002	emm6	M6	5	4	4	N/A	N/A	1	N/A
Haukness 2002	emm6	M6	6	6	N/A	N/A	N/A	N/A	N/A

N/A= Not available

Table S9. Summary of emm clusters (>2 studies) associated with the significant virulence factors.

Cluster	Emm type	No. of studies	No. of isolates	*Percentage of virulence factors (%)*					
				speA	*speG*	*speK*	*SpeM*	*ssa*	*smeZ*
AC3	emm1	17	908	91	72	1	1	17	73
AC4	emm12	16	747	11	71	1	1	37	71
AC5	emm3	12	272	96	88	28	10	71	62
D4	emm33, emm41, emm43, emm53, emm64, emm70, emm80, emm83, emm86, emm91, emm93, emm101, emm119, emm223	5	22	27	36	0	18	5	91
E1	emm4, emm78, emm165	14	379	7	22	1	2	88	70
E2	emm13, emm50, emm66, emm68, emm76, emm90, emm92, emm106	6	46	30	63	0	7	15	65
E3	emm9, emm25, emm44, emm49, emm58, emm82, emm87, emm183	8	97	23	74	0	3	42	60
E4	emm2, emm8, emm22, emm28, emm73, emm77, emm84, emm88, emm89, emm102, emm112	16	1096	11	76	8	2	7	74
E6	emm11, emm59, emm63, emm65, emm75, emm81, emm85, emm94, emm177	9	311	8	92	7	11	4	70
M6	emm6	11	163	28	45	37	12	10	50
M18	emm18	4	19	68	26	0	5	16	32

speM- significant in high-quality studies any method.
speG, smeZ, ssa- significant in high-quality WGS studies.
speA, speK- significant in high-quality PCR studies.

References

1. Nasser W, Beres SB, Olsen RJ, Dean MA, Rice KA, Long SW, et al. Evolutionary pathway to increased virulence and epidemic group A Streptococcus disease derived from 3,615 genome sequences. Proceedings of the National Academy of Sciences. 2014;111(17):E1768-E76.
2. Meisal R, Høiby E, Caugant D, Musser JM. Molecular characteristics of pharyngeal and invasive emm 3 Streptococcus pyogenes strains from Norway, 1988–2003. European journal of clinical microbiology & infectious diseases. 2010;29:31-43.
3. Al-Shahib A, Underwood A, Afshar B, Turner CE, Lamagni T, Sriskandan S, et al. Emergence of a novel lineage containing a prophage in emm/M3 group A Streptococcus associated with upsurge in invasive disease in the UK. Microbial genomics. 2016;2(6).
4. Vlaminckx BJ, Schuren FH, Montijn RC, Caspers MP, Beitsma M, Wannet WJ, et al. Dynamics in prophage content of invasive and noninvasive M1 and M28 Streptococcus pyogenes isolates in The Netherlands from 1959 to 1996. Infection and immunity. 2007;75(7):3673-9.
5. Rogers S, Commons R, Danchin MH, Selvaraj G, Kelpie L, Curtis N, et al. Strain prevalence, rather than innate virulence potential, is the major factor responsible for an increase in serious group A streptococcus infections. The Journal of infectious diseases. 2007;195(11):1625-33.
6. Vikerfors A, Haggar A, Darenberg J, Low A, Melhus Å, Hedlund J, et al. Severe group A streptococcal infections in Uppsala County, Sweden: clinical and molecular characterization of a case cluster from 2006 to 2007. Scandinavian journal of infectious diseases. 2009;41(11-12):823-30.
7. Åkesson P, Rasmussen M, Mascini E, Ulrich vP-R, Janulczyk R, Collin M, et al. Low antibody levels against cell wall-attached proteins of Streptococcus pyogenes predispose for severe invasive disease. Journal of Infectious Diseases. 2004;189(5):797-804.
8. Azuma K, Koike K, Kobayashi T, Mochizuki T, Mashiko K, Yamamoto Y. Detection of circulating superantigens in an intensive care unit population. International journal of infectious diseases. 2004;8(5):292-8.
9. Basma H, Norrby-Teglund A, Guedez Y, McGeer A, Low DE, El-Ahmedy O, et al. Risk factors in the pathogenesis of invasive group A streptococcal infections: role of protective humoral immunity. Infection and immunity. 1999;67(4):1871-7.
10. Yang LP, Eriksson BK, Harrington Z, Curtis N, Lang S, Currie BJ, et al. Variations in the protective immune response against streptococcal superantigens in populations of different ethnicity. Medical microbiology and immunology. 2006;195:37-43.
11. Arslan U, Oryaşın E, Eskin Z, Fındık D, Tuncer I, Bozdoğan B. Distribution of emm genotypes and antibiotic susceptibility of Streptococcus pyogenes strains: analogy with the vaccine in development. Mikrobiyoloji Bulteni. 2013;47(2):318-23.
12. Chalker V, Jironkin A, Coelho J, Al-Shahib A, Platt S, Kapatai G, et al. Genome analysis following a national increase in scarlet fever in England 2014. BMC genomics. 2017;18(1):1-10.
13. Leung TN, Hon K, Leung AK. Group A Streptococcus disease in Hong Kong children: an overview. Hong Kong Medical Journal. 2018.
14. O'Brien KL, Levine OS, Schwartz B, editors. The changing epidemiology of group A Streptococcus infections. Seminars in Pediatric Infectious Diseases; 1997: Elsevier.
15. Parks T, Elliott K, Lamagni T, Auckland K, Mentzer AJ, Guy R, et al. Elevated risk of invasive group A streptococcal disease and host genetic variation in the human leucocyte antigen locus. Genes & Immunity. 2020;21(1):63-70.
16. Sanson MA, Macias OR, Shah BJ, Hanson B, Vega LA, Alamarat Z, et al. Unexpected relationships between frequency of antimicrobial resistance, disease phenotype and emm type in group A Streptococcus. Microbial Genomics. 2019;5(11).

17. Kawabata S. Virulence factors in Streptococcus pyogenes infection. Nihon Saikingaku zasshi Japanese Journal of Bacteriology. 2005;60(2):365-74.
18. Newton D, Norrby-Teglund A, McGeer A, Low DE, Schlievert PM, Kotb M. Novel superantigens from streptococcal toxic shock syndrome Streptococcus pyogenes isolates. Streptococci and the Host. 1997:525-9.
19. Brown EJ. The molecular basis of streptococcal toxic shock syndrome. New England Journal of Medicine. 2004;350(20):2093-4.
20. Cherneski CL, Embil JM. Necrotizing fasciitis. Saudi medical journal. 2001;22(7):565-8.
21. Cole JN, Barnett TC, Nizet V, Walker MJ. Molecular insight into invasive group A streptococcal disease. Nature Reviews Microbiology. 2011;9(10):724-36.
22. Steer AC, Lamagni T, Curtis N, Carapetis JR. Invasive group a streptococcal disease: epidemiology, pathogenesis and management. Drugs. 2012;72:1213-27.
23. Vlaminckx B, Mascini E, Schellekens J. Invasive Lancefield group A streptococcal infections in the Netherlands. Nederlands Tijdschrift Voor Geneeskunde. 2007;151(30):1669-73.
24. Matsumoto M, Yamada K, Suzuki M, Adachi H, Kobayashi S, Yamashita T, et al. Description of the pathogenic features of Streptococcus pyogenes isolates from invasive and non-invasive diseases in Aichi, Japan. Japanese journal of infectious diseases. 2016;69(4):338-41.
25. Cleary PP, Schlievert P, Handley JP, Kim M, Hauser A, Kaplan E, et al. Clonal basis for resurgence of serious Streptococcus pyogenes disease in the 1980s. The Lancet. 1992;339(8792):518-21.
26. Friães A, Pinto FR, Silva-Costa C, Ramirez M, Melo-Cristino J, Infections PGftSoS. Group A streptococci clones associated with invasive infections and pharyngitis in Portugal present differences in emm types, superantigen gene content and antimicrobial resistance. BMC microbiology. 2012;12(1):280.
27. Bencardino D, Di Luca MC, Petrelli D, Prenna M, Vitali LA. High virulence gene diversity in Streptococcus pyogenes isolated in Central Italy. PeerJ. 2019;7:e6613.
28. Luca-Harari B, Straut M, Cretoiu S, Surdeanu M, Ungureanu V, van der Linden M, et al. Molecular characterization of invasive and non-invasive Streptococcus pyogenes isolates from Romania. Journal of medical microbiology. 2008;57(11):1354-63.
29. Bianco S, Allice T, Zucca M, Savoia D. Survey of phenotypic and genetic features of Streptococcus pyogenes strains isolated in Northwest Italy. Current microbiology. 2006;52:33-9.
30. Li Y, Dominguez S, Nanduri SA, Rivers J, Mathis S, Li Z, et al. Genomic Characterization of Group A Streptococci Causing Pharyngitis and Invasive Disease in Colorado, USA, June 2016–April 2017. The Journal of Infectious Diseases. 2022;225(10):1841-51.
31. Golińska E, Van der Linden M, Więcek G, Mikołajczyk D, Machul A, Samet A, et al. Virulence factors of Streptococcus pyogenes strains from women in peri-labor with invasive infections. European Journal of Clinical Microbiology & Infectious Diseases. 2016;35(5):747-54.
32. Jing H-b, Ning B-a, Hao H-j, Zheng Y-l, Chang D, Jiang W, et al. Epidemiological analysis of group A streptococci recovered from patients in China. Journal of medical microbiology. 2006;55(8):1101-7.
33. Kittang BR, Skrede S, Langeland N, Haanshuus CG, Mylvaganam H. emm gene diversity, superantigen gene profiles and presence of SlaA among clinical isolates of group A, C and G streptococci from western Norway. European journal of clinical microbiology & infectious diseases. 2011;30:423-33.
34. Chan JC, Chu Y-W, Chu M-Y, Cheung TK, Lo JY. Epidemiological analysis of Streptococcus pyogenes infections in Hong Kong. Pathology. 2009;41(7):681-6.
35. Coppens J, Xavier BB, Loens K, Lammens C, Ieven M, Matheeussen V, et al. Remarkable genome stability among emm1 group a Streptococcus in Belgium over 19 Years. Genome biology and evolution. 2019;11(5):1432-9.

36. Darenberg J, Luca-Harari B, Jasir A, Sandgren A, Pettersson H, Schalén C, et al. Molecular and clinical characteristics of invasive group A streptococcal infection in Sweden. Clinical infectious diseases. 2007;45(4):450-8.
37. Ekelund K, Darenberg J, Norrby-Teglund A, Hoffmann S, Bang D, Skinhøj P, et al. Variations in emm type among group A streptococcal isolates causing invasive or noninvasive infections in a nationwide study. Journal of clinical microbiology. 2005;43(7):3101-9.
38. Khan RMA, Anwar S, Pirzada ZA. Streptococcus pyogenes strains associated with invasive and non-invasive infections present possible links with emm types and superantigens. Iranian Journal of Basic Medical Sciences. 2020;23(1):133.
39. Maripuu L, Eriksson A, Norgren M. Superantigen gene profile diversity among clinical group A streptococcal isolates. FEMS Immunology & Medical Microbiology. 2008;54(2):236-44.
40. Meehan M, Murchan S, Gavin PJ, Drew RJ, Cunney R. Epidemiology of an upsurge of invasive group A streptococcal infections in Ireland, 2012–2015. Journal of Infection. 2018;77(3):183-90.
41. Michaelsen T, Andreasson I, Langerud B, Caugant D. Similar Superantigen Gene Profiles and Superantigen Activity in Norwegian Isolates of Invasive and Non-Invasive Group A Streptococci. Scandinavian journal of immunology. 2011;74(5):423-9.
42. Muhtarova A, Gergova R, Setchanova L, Mitov I. Distribution of super-antigens and toxins in Bulgarian invasive and non-invasive clinical isolates Streptococcus pyogenes. Acta Microb Bulgar. 2017;33:151-6.
43. Rivera A, Rebollo M, Miro E, Mateo M, Navarro F, Gurguí M, et al. Superantigen gene profile, emm type and antibiotic resistance genes among group A streptococcal isolates from Barcelona, Spain. Journal of medical microbiology. 2006;55(8):1115-23.
44. Hraoui M, Boubaker IB-B, Doloy A, Samir E, Redjeb SB, Bouvet A. Epidemiological markers of Streptococcus pyogenes strains in Tunisia. Clinical microbiology and infection. 2011;17(1):63-8.
45. Yu D, Liang Y, Lu Q, Meng Q, Wang W, Huang L, et al. Molecular Characteristics of Streptococcus pyogenes Isolated From Chinese Children With Different Diseases. Frontiers in Microbiology. 2021;12:722225.
46. Strus M, Heczko P, Golińska E, Tomusiak A, Chmielarczyk A, Dorycka M, et al. The virulence factors of group A streptococcus strains isolated from invasive and non-invasive infections in Polish and German centres, 2009–2011. European Journal of Clinical Microbiology & Infectious Diseases. 2017;36(9):1643-9.
47. Descheemaeker P, Van Loock F, Hauchecorne M, Vandamme P, Goossens H. Molecular characterisation of group A streptococci from invasive and non-invasive disease episodes in Belgium during 1993–1994. Journal of medical microbiology. 2000;49(5):467-71.
48. Murakami J, Kawabata S, Terao Y, Kikuchi K, Totsuka K, Tamaru A, et al. Distribution of emm genotypes and superantigen genes of Streptococcus pyogenes isolated in Japan, 1994–9. Epidemiology & Infection. 2002;128(3):397-404.
49. Creti R, Gherardi G, Imperi M, von Hunolstein C, Baldassarri L, Pataracchia M, et al. Association of group A streptococcal emm types with virulence traits and macrolide-resistance genes is independent of the source of isolation. Journal of medical microbiology. 2005;54(10):913-7.
50. DelVecchio A, Maley M, Currie BJ, Sriprakash KS. NAD-glycohydrolase production and speA and speC distribution in group A streptococcus (GAS) isolates do not correlate with severe GAS diseases in the Australian population. Journal of clinical microbiology. 2002;40(7):2642-4.
51. Haukness HA, Tanz RR, Thomson Jr RB, Pierry DK, Kaplan EL, Beall B, et al. The heterogeneity of endemic community pediatric group a streptococcal pharyngeal

isolates and their relationship to invasive isolates. The Journal of infectious diseases. 2002;185(7):915-20.
52. Mylvaganam H, Bjorvatn B, Osland A. Distribution and sequence variations of selected virulence genes among group A streptococcal isolates from western Norway. Apmis. 2000;108(11):771-8.
53. Nandi S, Chakraborti A, Bakshi D, Rani A, Kumar R, Ganguly N. Association of pyrogenic exotoxin genes with pharyngitis and rheumatic fever/rheumatic heart disease among Indian isolates of Streptococcus pyogenes. Letters in applied microbiology. 2002;35(3):237-41.
54. Tyler S, Johnson W, Huang J, Ashton F, Wang G, Low D, et al. Streptococcal erythrogenic toxin genes: detection by polymerase chain reaction and association with disease in strains isolated in Canada from 1940 to 1991. Journal of clinical microbiology. 1992;30(12):3127-31.
55. Hsueh P-R, Wu J-J, Tsai P-J, Liu J-W, Chuang Y-C, Luh K-T. Invasive group A streptococcal disease in Taiwan is not associated with the presence of streptococcal pyrogenic exotoxin genes. Clinical infectious diseases. 1998;26(3):584-9.
56. Schmitz F-J, Beyer A, Charpentier E, Normark BH, Schade M, Fluit AC, et al. Toxin-gene profile heterogeneity among endemic invasive European group A streptococcal isolates. The Journal of infectious diseases. 2003;188(10):1578-86.
57. Lintges M, van der Linden M, Hilgers R-D, Arlt S, Al-Lahham A, Reinert RR, et al. Superantigen genes are more important than the emm type for the invasiveness of group A Streptococcus infection. The Journal of infectious diseases. 2010;202(1):20-8.
58. Plainvert C, Dinis M, Ravins M, Hanski E, Touak G, Dmytruk N, et al. Molecular epidemiology of sil locus in clinical Streptococcus pyogenes strains. Journal of clinical microbiology. 2014;52(6):2003-10.
